# Targeted Phenotypic Screening in *Plasmodium falciparum* and *Toxoplasma gondii* Reveals Novel Modes of Action of Medicines for Malaria Venture Malaria Box Molecules

**DOI:** 10.1128/mSphere.00534-17

**Published:** 2018-01-24

**Authors:** Gowtham Subramanian, Meenakshi A. Belekar, Anurag Shukla, Jie Xin Tong, Ameya Sinha, Trang T. T. Chu, Akshay S. Kulkarni, Peter R. Preiser, D. Srinivasa Reddy, Kevin S. W. Tan, Dhanasekaran Shanmugam, Rajesh Chandramohanadas

**Affiliations:** a Pillar of Engineering Product Development, Singapore University of Technology and Design, Singapore; b Biochemical Sciences Division, CSIR National Chemical Laboratory, Pune, India; c Department of Microbiology and Immunology, National University of Singapore, Singapore; d Organic Chemistry Division, CSIR National Chemical Laboratory, Pune, India; e School of the Biological Sciences, Nanyang Technological University, Singapore; Indiana University School of Medicine

**Keywords:** MMV Malaria Box, malaria, *Plasmodium falciparum*, *Toxoplasma gondii*, apicoplast, chemical phenotyping, egress, flow cytometry, invasion, merozoites, tachyzoites, toxoplasmosis

## Abstract

The Malaria Box collection includes 400 chemically diverse small molecules with documented potency against malaria parasite growth, but the underlying modes of action are largely unknown. Using complementary phenotypic screens against *Plasmodium falciparum* and *Toxoplasma gondii*, we report phenotype-specific hits based on inhibition of overall parasite growth, apicoplast segregation, and egress or host invasion, providing hitherto unavailable insights into the possible mechanisms affected. First, the Malaria Box library was screened against tachyzoite stage *T. gondii* and the half-maximal effective concentrations (EC_50_s) of molecules showing ≥80% growth inhibition at 10 µM were determined. Comparison of the EC_50_s for *T. gondii* and *P. falciparum* identified a subset of 24 molecules with nanomolar potency against both parasites. Thirty molecules that failed to induce acute growth inhibition in *T. gondii* tachyzoites in a 2-day assay caused delayed parasite death upon extended exposure, with at least three molecules interfering with apicoplast segregation during daughter cell formation. Using flow cytometry and microscopy-based examinations, we prioritized 26 molecules with the potential to inhibit host cell egress/invasion during asexual developmental stages of *P. falciparum*. None of the inhibitors affected digestive vacuole integrity, ruling out a mechanism mediated by broadly specific protease inhibitor activity. Interestingly, five of the plasmodial egress inhibitors inhibited ionophore-induced egress of *T. gondii* tachyzoites. These findings highlight the advantage of comparative and targeted phenotypic screens in related species as a means to identify lead molecules with a conserved mode of action. Further work on target identification and mechanism analysis will facilitate the development of antiparasitic compounds with cross-species efficacy.

**IMPORTANCE** The phylum *Apicomplexa* includes many human and animal pathogens, such as *Plasmodium falciparum* (human malaria) and *Toxoplasma gondii* (human and animal toxoplasmosis). Widespread resistance to current antimalarials and the lack of a commercial vaccine necessitate novel pharmacological interventions with distinct modes of action against malaria. For toxoplasmosis, new drugs to effectively eliminate tissue-dwelling latent cysts of the parasite are needed. The Malaria Box antimalarial collection, managed and distributed by the Medicines for Malaria Venture, includes molecules of novel chemical classes with proven antimalarial efficacy. Using targeted phenotypic assays of *P. falciparum* and *T. gondii*, we have identified a subset of the Malaria Box molecules as potent inhibitors of plastid segregation and parasite invasion and egress, thereby providing early insights into their probable mode of action. Five molecules that inhibit the egress of both parasites have been identified for further mechanistic studies. Thus, the approach we have used to identify novel molecules with defined modes of action in multiple parasites can expedite the development of pan-active antiparasitic agents.

## Abstract (precis)

The phylum *Apicomplexa* includes many human and animal pathogens, such as *Plasmodium falciparum* (human malaria) and *Toxoplasma gondii* (human and animal toxoplasmosis). Widespread resistance to current antimalarials and the lack of a commercial vaccine necessitate novel pharmacological interventions with distinct modes of action against malaria. For toxoplasmosis, new drugs to effectively eliminate tissue-dwelling latent cysts of the parasite are needed. The Malaria Box antimalarial collection, managed and distributed by the Medicines for Malaria Venture, includes molecules of novel chemical classes with proven antimalarial efficacy. Using targeted phenotypic assays of *P. falciparum* and *T. gondii*, we have identified a subset of the Malaria Box molecules as potent inhibitors of plastid segregation and parasite invasion and egress, thereby providing early insights into their probable mode of action. Five molecules that inhibit the egress of both parasites have been identified for further mechanistic studies. Thus, the approach we have used to identify novel molecules with defined modes of action in multiple parasites can expedite the development of pan-active antiparasitic agents.

## INTRODUCTION

Malaria remains a major health burden globally, as exemplified by the 212 million reported new cases in 2015 alone, distributed across the WHO African region (90%), the WHO Southeast Asia region (7%), and the WHO eastern Mediterranean region (2%) (1). Morbidity and mortality resulting from malaria are primarily due to infections with *Plasmodium falciparum*, a species that tends to rapidly gain resistance against frontline antimalarial therapies (2, 3). Therefore, new antimalarial compounds, ideally based on novel chemical scaffolds and distinct mechanisms of action, are needed to buttress the existing antimalarial treatment options. As a way to address this, Medicines for Malaria Venture (MMV), Geneva, Switzerland, has coordinated the creation of the Malaria Box antimalarial collection of 400 nontoxic chemicals of natural and synthetic origins, consisting of 200 drug-like and 200 probe-like molecules (4). These molecules were originally identified by a high-throughput *in vitro* antimalarial screening campaign against asexual intraerythrocytic stages of *P. falciparum*, starting with thousands of compounds, in an effort based at the Saint Jude Children’s Research Hospital (United States) (4, 5), Novartis (United States) (6), and GlaxoSmithKline (United States, United Kingdom, and Spain) (7). Many of these compounds potently inhibit parasite growth *in vitro* with half-maximal effective concentrations (EC_50_s) ranging from low micromolar to nanomolar efficacy. Owing to the free availability of this collection to the malaria scientific community, a number of studies have been undertaken to decipher their effects on various parasitic developmental stages and modes of action and to establish their *in vivo* efficacy (8–13). Previous work has identified molecules capable of inhibiting coenzyme A synthesis (14), apicoplast function (15), and β-hematin formation (16). In addition, the bioactivity of the Malaria Box collection against other pathogens, such as *Schistosoma mansoni* cercariae (17), *Cryptosporidium parvum* (18), *Perkinsus marinus* (19), *Trypanosoma cruzi* (20), *Trypanosoma brucei*, *Leishmania donovani*, *Leishmania infantum* (21), *Leishmania major* (22), and *Echinococcus multilocularis* (23), and on cancer cell lines (24) has been determined.

There is considerable interest in identifying the target(s) and understanding the modes of action of Malaria Box molecules as an essential step toward carrying out further medicinal chemistry optimizations, resulting eventually in drug development. However, deciphering the mechanism and identifying the target are time-consuming and difficult processes. Recently, mass spectrometry-based metabolic perturbation profiling following inhibitor treatment was carried out by two groups (25, 26), covering more than half of the Malaria Box collection, to identify their likely mode of action on the basis of their broad metabolic effect. By far, the most successful approach used to identify antimalarial targets and address the mechanism of action/resistance of novel inhibitors is the generation of resistant parasites in the laboratory and identification of the specific mutations linked to the resistance phenotype (27). Despite these advances, the mode of action of most of the molecules in this collection remains unknown, making them perfect starting points to dissect new pathways in infection biology, to validate novel target discovery, and eventually facilitate the development of novel antimalarial drugs.

In this study, we have explored the phenotypic effects of Malaria Box molecules in two closely related parasitic species, *P. falciparum* and *Toxoplasma gondii*, to identify and exploit molecules that have complementary inhibitory effects on both species. Because of their shared evolutionary history of these two parasites, a large percentage of the genes they carry, 2,482 *P. falciparum* genes (~50% of the total) and 2,591 *T. gondii* genes (~30% of the total), are orthologous (28, 29). Many of these conserved genes make up core components of indispensable cellular processes required by both parasites for development, replication, egress, or invasion. Hence, it is reasonable to expect comparable phenotypic responses upon exposure to a molecule that might target orthologous proteins. As a precedent, for example, antibiotics affecting plastid housekeeping functions by targeting orthologous proteins were shown to have similar phenotypic effects on *P. falciparum* and *T. gondii* (30–34). Furthermore, the apical organelles, such as the rhoptries and micronemes, and many of the resident proteins in these compartments involved in invasion are orthologous and conserved in these parasites (35–37). Inhibitors targeting these proteins are likely to be effective against both parasites. For example, perforins and proteases (38, 39), which help in active egress of the parasite from infected host cells, are conserved in both *P. falciparum* and *T. gondii*. Moreover, despite differences in the host cell type specificity of these parasites, they appear to co-opt the same host factors to induce host cytolysis and egress (40), highlighting deeply conserved similarities at the molecular level.

We present here results of a systematic study on phenotypic screening of the MMV Malaria Box compounds against *T. gondii* and *P. falciparum*. Of a total of 390 molecules, 136 were found to significantly affect *T. gondii* growth and 24 of these hits had nanomolar potency against both parasites. Of a subset of 30 molecules that caused “delayed death” of *T. gondii*, 3 were found to act through a mechanism involving plastid missegregation during daughter cell formation. Using flow cytometry assays designed to monitor the schizont-to-ring transition of intraerythrocytic stages of *P. falciparum*, 26 molecules were shortlisted on the basis of their ability to either block schizont maturation and host cell rupture or inhibit host cell invasion by extracellular merozoites. These molecules were further validated in secondary assays to ascertain their specificity as egress or invasion inhibitors. None of the egress or invasion blockers identified in the Malaria Box collection had any effect on *P. falciparum* digestive vacuole (DV) physiology. Cross-species testing of *P. falciparum* egress inhibitors identified five molecules as potent blockers of ionophore-mediated egress of *T. gondii* tachyzoites. Taken together, our findings provide compelling evidence of how conserved phenotypic effects on two related parasites can help in prioritizing molecules from the MMV Malaria Box collection for further mechanistic studies and the development of novel antiparasitic compounds.

## RESULTS

### Overview of targeted phenotype screening of Malaria Box molecules against *P. falciparum* and *T. gondii*.

**FIG 1:**
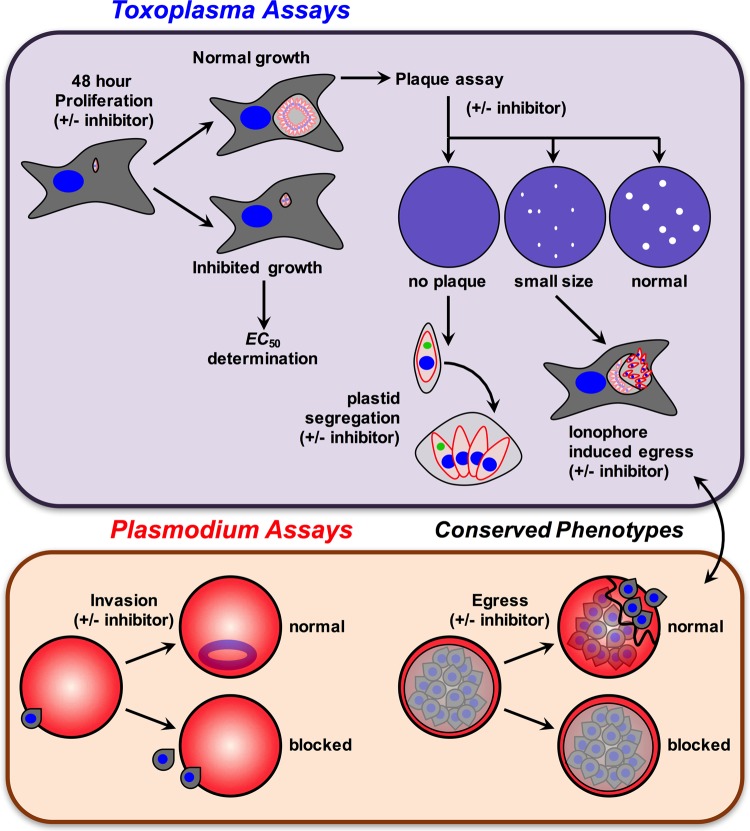
Overview of the phenotypic assays used for screening of the MMV Malaria Box library against *P. falciparum* and *T. gondii*.

In this study, we characterized 390 molecules belonging to the MMV Malaria Box collection on the basis of the phenotypic effects they induce in two evolutionarily related apicomplexan parasites, *P. falciparum* and *T. gondii*. We reasoned that such an approach will help to identify parasite-specific, as well as cross-species, effects of these molecules during life cycle stages that are relevant to disease progression and clinical outcome, such as the asexual intraerythrocytic stage of *P. falciparum* and the tachyzoites stage of *T. gondii*. To do this, we undertook a multipronged yet targeted phenotype screening approach, as schematically depicted in Fig. 1. Before embarking on the phenotypic studies, it was important to derive the bioactivity of all Malaria Box compounds in both species. For this, we first determined the in-house EC_50_s of all MMV Malaria Box molecules against *P. falciparum* and *T. gondii* by using the compound library received from MMV.

### Comparing the efficacies of Malaria Box library compounds against *P. falciparum* and *T. gondii*

**FIG 2:**
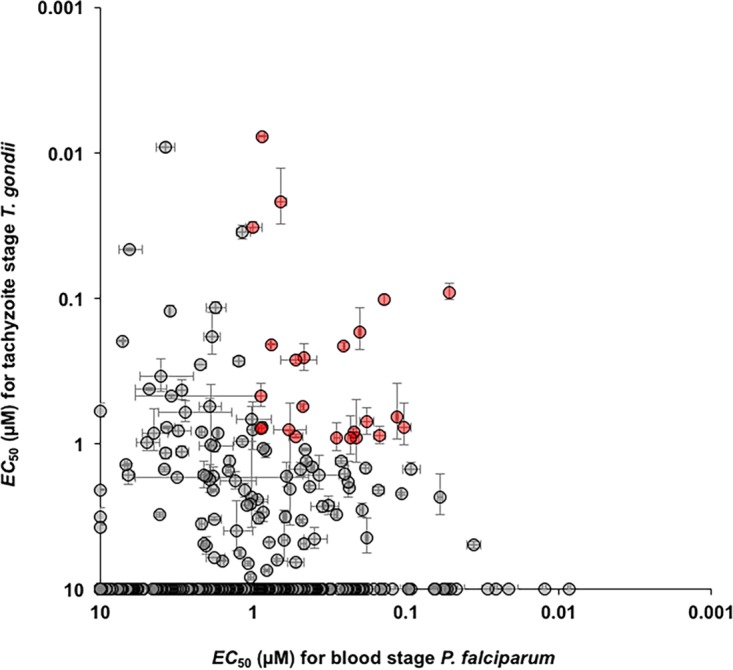
Correlation of the overall efficacy (EC_50_) of the MMV Malaria Box library against *P. falciparum* and *T. gondii*. The inhibitory potencies of Malaria Box molecules against asexual blood stage *P. falciparum* and tachyzoite stage *T. gondii* were compared. The 24 molecules with nanomolar potency against both parasites are highlighted in red. Molecules whose EC_50_s were not determined (those with <80% growth inhibition at 10 µM) are plotted at 10 µM EC_50_ for the respective parasites. Average values of three replicates are plotted, and error bars indicate standard deviations.

For EC_50_ determination in *Plasmodium*, sorbitol-synchronized ring stage parasites (strain 3D7) were treated with 2-fold serial dilutions of the molecules ranging from 10 µM to ~5 nM. Following this, parasitemia was scored at ~60 h postinfection (hpi) by SYBR green I staining of parasitic DNA. As expected, nearly 50% of the molecules showed nanomolar efficacy against *P. falciparum*, in good agreement with previous results (24). In parallel, 48-h killing assays were carried out with transgenic *T. gondii* tachyzoites (type I RH strain) expressing firefly luciferase. The antitoxoplasma activity of Malaria Box molecules was estimated by comparing the parasitemia levels to 1% dimethyl sulfoxide (DMSO) control- and inhibitor-treated parasites with a standardized luminescence readout assay (see Fig. S1 in the supplemental material). Of the total of 390 molecules tested, 136 inhibited *T. gondii* growth by >75% at 10 µM. Importantly, 49 of these compounds showed nanomolar potency (Table S1), in contrast to a previous report (41). We then compared the EC_50_s for *P. falciparum* and *T. gondii* and identified 24 molecules with nanomolar potency against both parasites (Fig. 2; Table S1).

10.1128/mSphere.00534-17.1 FIG S1  Optimization of luminescence assay parameters for RH-Luc transgenic parasites. (A) Checking the linearity of luminescence detection with extracellular RH-Luc tachyzoite stage parasites. A 2-fold serial dilution, starting with 5 × 10^3^ parasites, was plated in a 50-µl total volume in a 96-well plate. A 50-µl volume of 2× luciferase assay reagent (Promega) was then added and mixed well. The plates were immediately subject to luminescence reading with a VarioScan plate reader (Thermo Fisher, United States). The samples were tested in triplicate, and the raw data were analyzed and plotted with the Excel spreadsheet program (Microsoft). Samples were analyzed in triplicate. (B) Detection of luminescence readout following 48 h of proliferation starting with various numbers of inoculated parasites. With a total culture volume of 200 µl/well, 10^3^, 5 × 10^3^, or 10^4^ parasites were inoculated into confluent HFF monolayers grown in 96-well plates. After 48 h of growth under optimal conditions, 150 µl of culture medium was removed and a luciferase assay was carried out as described above. Download FIG S1, PDF file, 0.05 MB. Copyright © 2018 Subramanian et al. 2018 Subramanian et al. This content is distributed under the terms of the Creative Commons Attribution 4.0 International license.

10.1128/mSphere.00534-17.10 TABLE S1  Compilation of efficacy and phenotypic screens of the MMV Malaria Box library against *P. falciparum* and *T. gondii*. Download TABLE S1, XLSX file, 0.1 MB. Copyright © 2018 Subramanian et al. 2018 Subramanian et al. This content is distributed under the terms of the Creative Commons Attribution 4.0 International license.

### Identification of molecules causing delayed death in *T. gondii*.

In the 48-h killing assays, 191 molecules from the Malaria Box collection showed only moderate inhibition of *T. gondii* tachyzoite growth (<50% inhibition at 10 µM; Fig. S2; Table S1). Of these, 134 molecules had virtually no effect on parasite growth (<20% inhibition at 10 µM). We were interested in evaluating if any of these molecules induce delayed death in *T. gondii* tachyzoites. Delayed death of apicomplexan parasites is a well-studied phenotype for antibiotics such as chloramphenicol, which targets the ribosomal machinery in the apicoplast (42–44). In this case, parasites continue to replicate within the first parasitophorous vacuole (PV) following inhibitor treatment and exhibit no apparent growth inhibition until they egress and reinvade a naive host cell, where they fail to replicate and die in the second vacuole. The molecular mechanism behind this delayed-death phenomenon appears to be associated with inhibition of housekeeping functions in the apicoplast (32, 45–47).

10.1128/mSphere.00534-17.2 FIG S2  Profiling of the activity of Malaria Box molecules against tachyzoite stage *T. gondii*. (A) Determination of the percent growth inhibition activity of the complete Malaria Box collection in a 48-h proliferation assay with the RH-Luc parasite line. The pink-shaded region highlights 119 molecules with >80% growth inhibition whose EC_50_s were determined. The blue-shaded region represents the 134 molecules with <20% growth inhibition that were subject to delayed-death and apicoplast missegregation assays. (B) The EC_50_s of 119 molecules with >80% growth inhibition were determined, and 49 were found to have nanomolar (<1 µM) potency. The methodology used for antitoxoplasma bioactivity profiling is discussed in Materials and Methods. The corresponding percent growth inhibition values and EC_50_s of the molecules are shown in Table S1. Download FIG S2, PDF file, 0.1 MB. Copyright © 2018 Subramanian et al. 2018 Subramanian et al. This content is distributed under the terms of the Creative Commons Attribution 4.0 International license.

**FIG 3:**
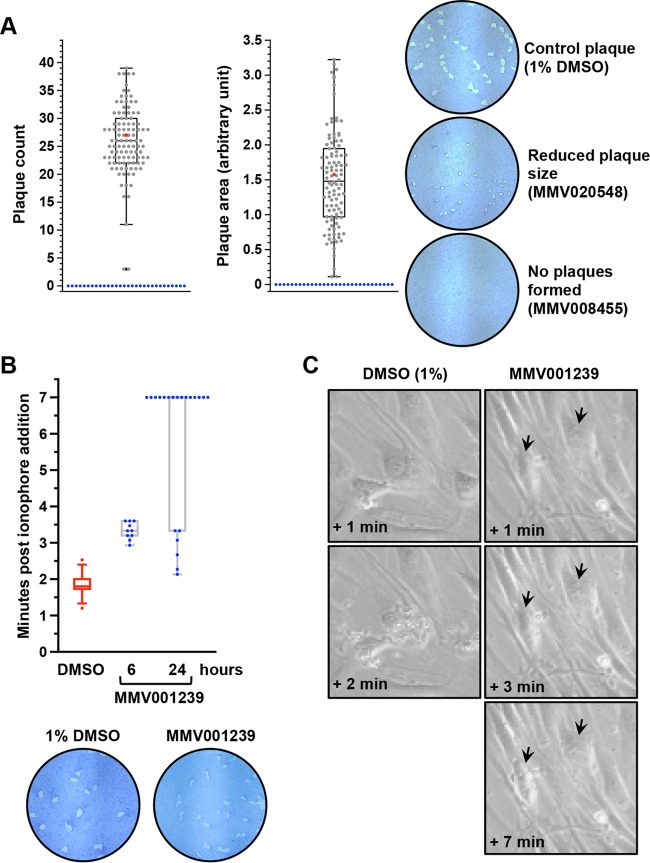
Plaque-forming assays identify compounds that cause delayed death of *T. gondii* and inhibit its egress. (A) From plaque assays, the number of plaques formed (plaque count plot) and the sizes of the plaques formed (plaque size plot) were determined. Average values for 1% DMSO-treated control samples are plotted in red. Thirty molecules (blue) were found to totally abolish plaque formation. Representative plaque images are shown for the control and two inhibitors that either reduced plaque size or completely inhibited plaque formation. (B) Inhibition of ionophore-induced egress by MMV001239, with a median egress time of >3 min and >10 min for 6 and 24 h of treatment with the compound at 10 µM, respectively. The median egress time of 1% DMSO-treated control parasites is 1.8 min. At the bottom are the plaques with reduced size (1.083 U) formed in the presence of MMV001239 in comparison with 1% DMSO-treated controls (2.23 U). (C) Time-lapse microscopic images showing a delay in ionophore-induced parasite egress following 24 h of treatment with MMV001239. Arrows indicate PVs.

To evaluate possible delayed-death effects of the 134 Malaria Box molecules that showed no significant effects on *T. gondii* in 48-h killing assays, we allowed tachyzoite stage parasites to form plaques (zones of host cell lysis formed by multiple rounds of invasion and egress by parasites) on confluent host cell monolayers in the presence of inhibitors. Confluent human foreskin fibroblast (HFF) monolayers in six-well plates were inoculated with 50 parasites per well for plaque formation. Molecules were plated at 10 µM, and 1% DMSO-treated cells were used as controls. The infected cultures were left undisturbed for 10 days, after which they were processed for visualization of plaque formation. The total number of plaques formed per well and the average plaque area were quantified for each treatment. A spectrum of growth phenotypes with respect to plaque number and size was obtained in the plaque assays. Most of the molecules tested did not significantly affect the number of plaques formed with respect to DMSO-treated control cultures and averaged between 20 and 35 plaques per well (Fig. 3A). In the case of two molecules (MMV666597 and MMV665882), we observed only three plaques each and these were dramatically reduced in size (~0.1 U compared to a control plaque size of ~1.58 U). For many other inhibitors, even though the plaque counts were similar to those of control cultures, the plaque size was reduced to at least half of the control size (Fig. 3A; Table S1). This can be due to either altered intracellular growth and replication of the parasite in the presence of the inhibitor or a delay in parasite egress from infected host cells, resulting in delayed progress in plaque formation.

We selected 21 molecules that caused a reduction in plaque size (for example, MMV020548, as shown in Fig. 3A, right panel) to test their inhibitory effects on *T. gondii* egress. For this, we adopted the widely used assay in which the calcium ionophore A23187 is used to induce intracellular tachyzoite egress (48) and monitored the time delay before the rupture of individual vacuoles and parasite release. Parasites were allowed to infect and replicate in HFF monolayers in the presence of either 10 µM inhibitor (or 1% DMSO as a negative control) for 24 h, followed by ionophore addition to induce egress. The time required for parasite egress from individual vacuoles was recorded and compared between DMSO- and inhibitor-treated parasites. The average egress time obtained for 1% DMSO-treated control parasites was ~1.8 min. In the case of inhibitor-treated parasites, we looked for an average egress time of at least 4 min to identify potential egress inhibitors. Of the 21 molecules tested, 20 had no significant inhibitory effect on ionophore-induced parasite egress (Fig. S3), suggesting that the reduced plaque size observed in the presence of these molecules is primarily due to diminished parasite fitness rather than a delay in parasite egress. However, one molecule, MMV001239, significantly inhibited parasite egress (Fig. 3B and C). It is interesting that MMV001239 exhibited time-dependent egress-blocking activity, as the delay in egress was much less pronounced upon treatment with 10 µM compound for 6 h (average egress time, ~3.3 min) than upon treatment for 24 h, where parasites failed to egress from most of the vacuoles even after 10 min following ionophore treatment (Fig. 3B).

10.1128/mSphere.00534-17.3 FIG S3  Profiling of the egress inhibition activity of selected Malaria Box molecules. Twenty Malaria Box molecules that were found to reduce the size of plaques formed by *T. gondii* tachyzoites were tested for the ability to affect parasite egress following induction with the calcium ionophore A23187 (see the text for protocol details). Red, 1% DMSO-treated controls having a median egress time of ~1.8 min. Download FIG S3, PDF file, 0.04 MB. Copyright © 2018 Subramanian et al. 2018 Subramanian et al. This content is distributed under the terms of the Creative Commons Attribution 4.0 International license.

The egress inhibitory effect of MMV001239 on *T. gondii* appears to be very specific, as this molecule has no effect on parasite growth, and plaque assays revealed the formation of plaques slightly smaller than those of the untreated control (Fig. 3C). Reduced plaque size may result from delayed egress of parasites following each round of infection during the course of plaque formation. Thus, it appears that MMV001239 is a genuine inhibitor of egress in *T. gondii*. Interestingly, in a recent study, it was shown that MMV001239 targets the lanosterol-14-α-demethylase enzyme in *T. cruzi* (49). Even though *T. gondii* does not encode an orthologue of this enzyme, it is likely that MMV001239 acts by interfering with membrane lipid dynamics, which is known to facilitate parasite egress from host cells. Although MMV001239 may not be useful as an antitoxoplasma agent because of its inability to abrogate parasite growth, we expect that further mechanistic studies with this molecule can help in dissecting the parasite egress pathway and identifying novel targets.

### Effect of delayed-death molecules on apicoplast segregation in *T. gondii*

Interestingly, 30 Malaria Box molecules that had no effect of *T. gondii* growth in the killing assays were found to completely abolish plaque formation because of delayed death of the parasites. A well-documented phenotypic consequence associated with the delayed-death phenomenon is a failure to properly segregate the apicoplast organelle to newly forming daughter cells during cell division. This results in a PV containing a mixture of daughter cells with and without the apicoplast organelle. This phenotype has also been validated by genetic intervention (45). The plastidless parasites survive until they egress and invade a new host cell, where they die. This phenotype can be tracked microscopically by identifying PVs containing plastidless parasites.

**FIG 4:**
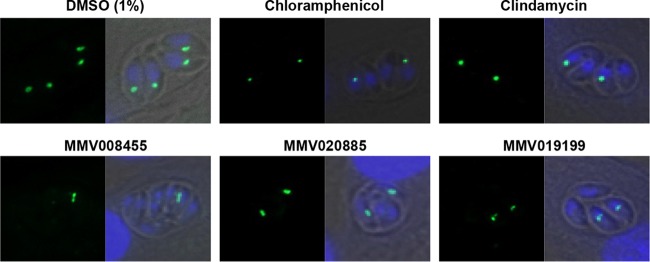
Plastid missegregation associated with the delayed death of *T. gondii* tachyzoites. Top panel, plastid missegregation phenotype observed in the second vacuole following treatment with chloramphenicol and clindamycin. Normal plastid segregation is evident in a 1% DMSO-treated control. At the bottom are representative microscopic images showing plastid missegregation for the Malaria Box molecules causing delayed death of the parasite.

We tested whether plastid missegregation occurs in *T. gondii* upon treatment with the 30 molecules that were found to completely abolish plaque formation. For this, we first allowed the parasites to invade and replicate within HFF monolayers in the presence of 10 µM inhibitor for 24 h. Parasites were then physically egressed, and host cell-free parasites were allowed to invade a naive confluent monolayer of HFF cells grown on glass coverslips in the continued presence of 10 µM inhibitor. Twenty-four hours later, the glass coverslips were fixed with 4% paraformaldehyde and processed for microscopy. In this experiment, a transgenic parasite line expressing the apicoplast-located triose phosphate isomerase II gene tagged with yellow fluorescent protein (YFP; *TgtpiII-yfp*) (Fig. S4) was used to visualize the apicoplast by the associated fluorescence. Among the 30 Malaria Box molecules tested in this assay, we observed an unambiguous plastid segregation defect in 3, MMV008455, MMV020885, and MMV019199, and a partial effect in 1, MMV666109 (Fig. 4). Chemically, these molecules are distinct from antibiotics such as chloramphenicol and clindamycin (Fig. S5), which are known to cause plastid missegregation and delayed death in apicomplexan parasites, and provide new tools to dissect this phenomenon in greater detail.

10.1128/mSphere.00534-17.4 FIG S4  Generation of the RH-*Tgtpi-II-yfp* transgenic parasite line. (A) Schematic diagram indicating the homologous recombination-mediated genomic locus tagging of the *Tgtpi-II* gene with a YFP tag at the 3′ end of the gene. The tagging construct also contains the DHFR selection cassette, which allows selection of transgenic lines with pyrimethamine. The asterisks depict the stop codon. The primer sequence used to amplify the genomic fragments and verify the insertion are shown as arrows, and their sequences are as follows: F1, 5′ GATCGTAAGCTTAGTCTTGAGTGAACAGCTTGAGGTAC 3′; F2, 5′ CGAAGAAAGTGCGCGCGGCGCTCAAC 3′; R1, 5′ GTCGTACCTAGGGGCTTGCTGCTTCGCTGCATCAATG 3′; R2, 5′ CCATGATATAGACGTTGTGGCTGTTGTAG 3′; R3, 5′ TCTAGAACTAGTGGATCCCCCTCCACC 3′. (B) Genomic PCR confirmation for proper tagging of YFP at the genomic locus of interest. Lanes 1, 2, 1′, and 2′ show PCR fragments that are expected from both the parental and transgenic *T. gondii* RH strains. Lanes 3, 4, 3′, and 4′ show the presence of tag-specific PCR products expected to be obtained only from the RH-*Tgtpi-II-yfp* transgenic parasite line. Lane L, 1-kb marker DNA ladder. Download FIG S4, PDF file, 0.2 MB. Copyright © 2018 Subramanian et al. 2018 Subramanian et al. This content is distributed under the terms of the Creative Commons Attribution 4.0 International license.

10.1128/mSphere.00534-17.5 FIG S5  Chemical structures of Malaria Box molecules causing apicoplast missegregation in *T. gondii*. Shown are the chemical structures of the respective Malaria Box molecules highlighting their distinction from chloramphenicol and clindamycin, two antibiotics previously known to cause plastid missegregation in *T. gondii*. Download FIG S5, PDF file, 0.1 MB. Copyright © 2018 Subramanian et al. 2018 Subramanian et al. This content is distributed under the terms of the Creative Commons Attribution 4.0 International license.

### Effects of Malaria Box molecules on *P. falciparum* egress.

**FIG 5:**
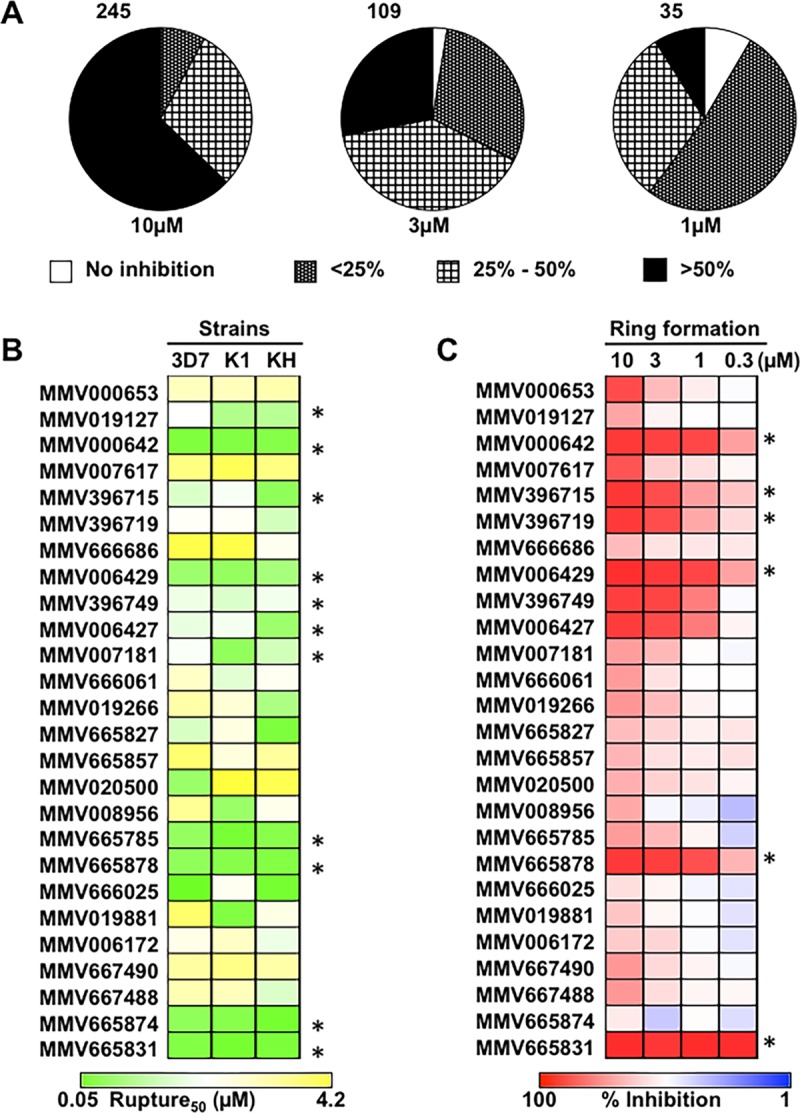
Identification of potential *P. falciparum* egress inhibitors in the Malaria Box library. (A) Pie charts showing the fraction of Malaria Box molecules showing <25, 25 to 50, and >50% inhibition of the schizont-to-ring transition in *P. falciparum* at 1, 3, and 10 μM, respectively. The data are mean values of replicates in each of three independent experiments. Following validation by microscopy on Giemsa-stained smears, 26 compounds that showed consistent inhibition were selected for further experiments. (B) The 26 compounds selected were screened against three *P. falciparum* strains, 3D7 (wild-type laboratory strain), K1 (chloroquine resistant), and KH004-003 (artemisinin resistant), by flow cytometry-based counting of ring stage infections after inhibitor treatment. Molecules with nanomolar R_50_ values across all three strains are marked with asterisks. (C) Late-stage schizonts (~45 hpi) incubated with the 26 inhibitors were monitored for iRBC rupture and ring formation by flow cytometry. The percentages of the population in the ring stage at ~55 hpi in the presence of inhibitors at 10, 3, 1, and 0.3 µM are shown as a heat map. Asterisks indicate potent molecules, i.e., >50% inhibition even at a 0.3 µM inhibitor concentration.

Using a complementary approach combining flow cytometry and microscopy as previously reported (50, 51), we monitored the effects of Malaria Box molecules on late intraerythrocytic stages of *P. falciparum* (i.e., >40-hpi schizonts) with the goal of identifying inhibitors of egress and/or invasion. In the primary screen, schizont stage parasites (~40 to 42 hpi) were treated with 1, 3, and 10 μM inhibitors and allowed to develop until the appearance of ring stage parasite in DMSO-treated controls (~60 hpi, approximately 12 h postrupture). Parasitemia was scored by flow cytometry as described previously (50, 52). In parallel, microscopic examination of Giemsa-stained thin smears was performed following inhibitor treatment. E-64, cycloheximide, trichostatin A, and heparin were included as positive controls in all egress and invasion studies. At 10 μM, a large fraction (~62%) of the Malaria Box compounds inhibited the transition of schizonts to rings with ≥50% efficiency compared to that of DMSO-treated control samples. However, the observed effects of some of the molecules could be attributed to overall toxicity owing to the high drug concentration (10 µM) used for preliminary screening. At lower concentrations of 3 and 1 µM, the number of molecules that blocked the transition of schizonts to rings with ≥50% efficacy dropped to 109 and 35, respectively (Fig. 5A). Results of three independent flow cytometry experiments in combination with analysis of microscopic phenotypes allowed us to shortlist 26 molecules that appeared to affect the schizont-to-ring transition with a minimum of 50% inhibition efficacy (Table S1).

On the basis of these results, we shortlisted 26 molecules for comparison of their effects on the schizont-to-ring transition in three strains of malaria parasites, 3D7, K1, and KH004-003; K1 and KH004-003 are resistant to the frontline antimalarials chloroquine and artemisinin, respectively. In these parasites, we estimated the 50% rupture (R_50_) values, which were determined as the concentrations at which a 50% reduction in the newly formed ring population (compared to that of DMSO-treated controls) was observed. After inhibitor treatment (20 h later), the fraction of rings that remained in the culture was estimated by flow cytometry and used to calculate the R_50_ value. Of the 26 molecules, MMV019127, MMV000642, MMV396715, MMV006429, MMV396749, MMV006427, MMV007181, MMV665785, MMV665878, MMV665874, and MMV665831 inhibited the schizont-to-ring transition with nanomolar potency (R_50_, ≤500 nM) across all three of the strains tested (Fig. 5B; Fig. S6 to S8). Notably, the EC_50_ and R_50_ values were not always in concordance, and therefore there appears to be no direct correlation between overall killing potency and schizont-to-ring transition inhibition. For instance, MMV665831 showed comparable potency in both parasite killing and inhibition of the schizont-to-ring transition. In contrast, molecules with nanomolar EC_50_s, i.e., MMV000653, MMV007617, and MMV665857, blocked the schizont-to-ring transition by ≥50% only at severalfold higher concentrations. A third group of hits, MMV000642 and MMV006427, appeared to be more potent in blocking the schizont-to-ring transition than in parasite killing, exhibiting nanomolar R_50_ values, despite an overall EC_50_ in the micromolar range. Collectively, these observations point toward distinct mechanisms by which the molecules of interest seem to affect the egress/invasion of *P. falciparum* and subsequent progression of the lytic cycle.

10.1128/mSphere.00534-17.6 FIG S6  Flow cytometry plots and representative Giemsa images of concentration-dependent inhibition of host cell rupture assays with *P. falciparum* strain 3D7. Tightly synchronized late-stage parasites (40 to 42 hpi) were treated with 26 potent MMV Malaria Box compounds at concentrations ranging from 10 μM to 100 nM for 12 to 15 h before monitoring of the transition from the schizont stage to the ring stage. (Top) Representative flow cytometry data showing the percentages of invasion and ring formation following inhibitor treatment of strain 3D7. The ring population was gated on the basis of the profile of DMSO-treated control parasites. E-64 and trichostatin A served as positive controls for inhibition of stage transition. (Bottom) Estimation of dose-dependent effect on ring stage parasite formation to derive R_50_ values. Download FIG S6, PDF file, 0.7 MB. Copyright © 2018 Subramanian et al. 2018 Subramanian et al. This content is distributed under the terms of the Creative Commons Attribution 4.0 International license.

10.1128/mSphere.00534-17.7 FIG S7  Flow cytometry plots and representative Giemsa images of concentration-dependent inhibition of host cell rupture assays with chloroquine-resistant *P. falciparum* strain K1. See the legend to Fig. S6. Download FIG S7, PDF file, 0.6 MB. Copyright © 2018 Subramanian et al. 2018 Subramanian et al. This content is distributed under the terms of the Creative Commons Attribution 4.0 International license.

10.1128/mSphere.00534-17.8 FIG S8  Flow cytometry plots and representative Giemsa images of concentration-dependent inhibition of host cell rupture assays with artemisinin-resistant *P. falciparum* strain KH004-003. See the legend to Fig. S6. Download FIG S8, PDF file, 0.5 MB. Copyright © 2018 Subramanian et al. 2018 Subramanian et al. This content is distributed under the terms of the Creative Commons Attribution 4.0 International license.

Next, we carried out a more detailed analysis of the egress/invasion inhibition effect of the 26 Malaria Box molecules selected from the initial screen. Magnetically purified schizonts (~40 to 42 hpi) were added to fresh red blood cells (RBCs) and incubated in the presence of inhibitors at 0.3, 1, 3, and 10 μM. Samples were collected 20 h later to score unruptured schizonts and newly invaded ring stage infections by flow cytometry. DMSO- and E-64-treated parasites served as negative and positive controls, respectively. Several molecules, i.e., MMV000642, MMV396715, MMV396719, MMV006429, MMV665878, and MMV665831, were able to block infected RBC (iRBC) rupture effectively at concentrations as low as 0.3 μM (Fig. 5C). The inhibitory potential of MMV665831 was profound; with an almost 90% reduction of ring formation across all of the concentrations tested. A >60% reduction of ring formation was observed at 0.3 µM with the molecules MMV000642, MMV396715, MMV006429, and MMV665878.

### Distinguishing egress and invasion inhibition activities.

**FIG 6:**
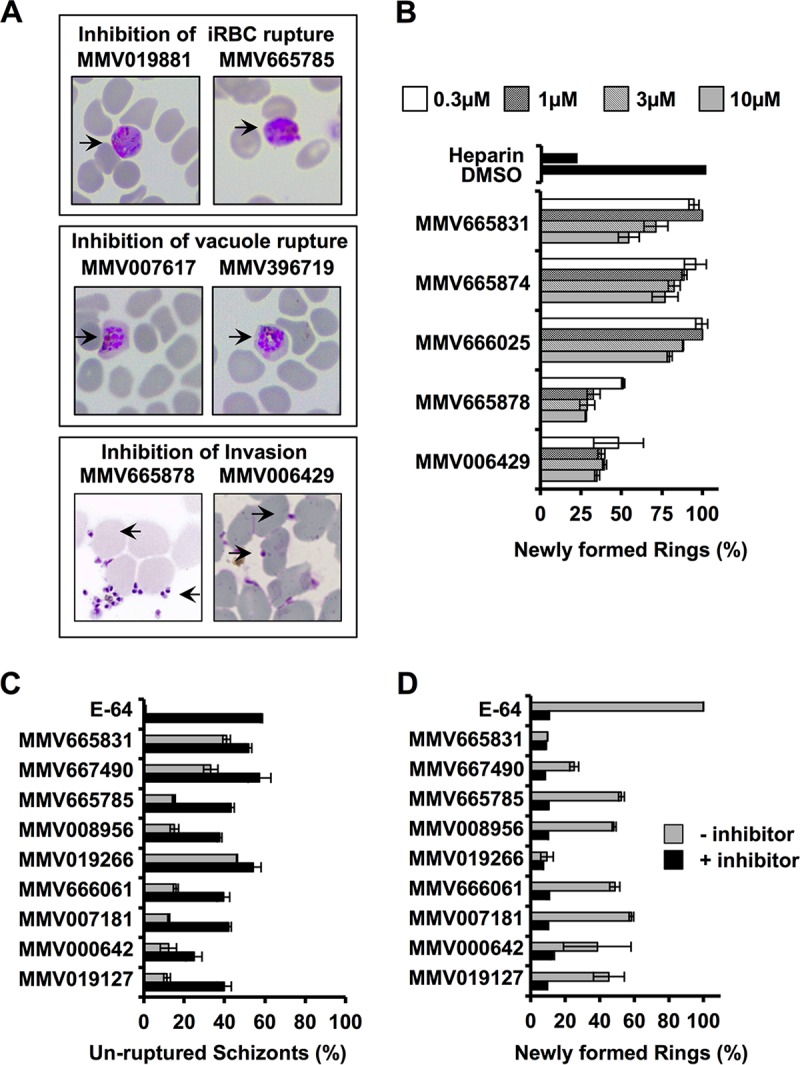
Egress/invasion phenotypic characterization of selected hits. (A) Giemsa-stained smears of inhibitor-treated parasites (all treated at 3 µM) exhibit previously reported phenotypes indicative of inhibition of iRBC membrane rupture (top), inhibition of PV membrane rupture (middle), and block of host cell invasion (bottom). (B) Invasion assays were carried out with molecules that appeared to interfere with merozoite invasion, as observed via microscopy. MMV665878 and MMV006429 were found to have potent inhibitory activity on extracellular merozoites. Inhibitor washing was performed to evaluate the reversibility of egress inhibition by testing for either a maturation block in schizonts (C) or a lack of ring stage formation (D) at ~55 hpi. The inhibitory effects of all of the molecules, except MMV665831, MMV667490, and MMV019266, were reversible by washing. Average values of three replicates are plotted, and error bars indicate standard deviations.

Since the flow cytometry experiments used to identify potential egress/invasion inhibitors cannot distinguish between the two types of mechanistically distinct molecules, we resorted to microscopic examination following treatment with selected inhibitors. This was done to segregate the molecules as specifically egress or invasion blockers. We examined representative Giemsa-stained smears postrupture (timing of rupture estimated from DMSO-treated control parasites) to distinguish the phenotypes associated with impaired late-stage parasite development. We found that following treatment with MMV019881 and MMV665785, merozoites were trapped within iRBCs, even though the PV appeared to have been dissolved, at 7 h after the rupture time point (~55 hpi) (Fig. 6A, top). In contrast, MMV007617 and MMV396719 induced a phenotype wherein merozoites appeared to be arrested within the PV (Fig. 6A, middle) inside intact iRBCs. Interestingly, when schizonts were incubated with MMV665878 or MMV006429, merozoite release was unaffected but merozoites were found attached to the surface of RBCs (Fig. 6A, bottom). This phenotype is typical of invasion inhibitors and is reminiscent of that previously reported for heparin, a proven invasion inhibitor (53, 54).

Five of the molecules that appeared to interfere with invasion (MMV006429, MMV665878, MMV666025, MMV665874, and MMV665831) were chosen for invasion studies with isolated merozoites. Late-stage schizonts (~46 to 48 hpi) purified by magnet-activated cell sorting (MACS) were physically ruptured by passage through a 1.2-μm filter as previously reported (55). After removal of the debris, noninfected RBCs and unruptured iRBCs were separated by differential centrifugation and merozoites were collected and added to fresh RBCs in the presence of serial dilutions of inhibitors. Heparin and DMSO served as positive and negative controls for invasion inhibition, respectively. Cultures were sampled for microscopy and flow cytometry after 4 and 15 h of incubation, respectively. MMV006429 and MMV665878 showed profound merozoite invasion inhibitory activity (~50%) at concentrations as low as 0.3 μM (Fig. 6B). The reduction in the number of ring stage-infected cells (relative to DMSO-treated control cells) confirms the invasion inhibitory activity of these two molecules. This was again validated by microscopy, where merozoites were found attached to the RBC surface as typically observed when invasion is impaired.

Merozoite egress is mediated by a variety of factors, including proteolytic activities (56, 57) that compromise host membrane architecture and changes in iRBC permeability facilitated through pore-forming proteins (38) in addition to progressive biophysical and ultrastructural changes (58). Proteases involved in egress are triggered in a time-dependent fashion, with their active forms appearing only at the right time to facilitate membrane rupture and release of merozoites. In contrast, other molecular effectors of egress either remain throughout or progressively build up during schizogony, leading to iRBC rupture. It is apparent that the molecules affecting different stages of egress act through distinct mechanisms and therefore egress blockage at distinct stages will manifest as distinct phenotypes.

To evaluate the timing and specificity of egress inhibition in *P. falciparum*, we selected the top nine egress inhibitors from our screens and treated the parasites with these molecules for a brief period before washing them away and allowing the parasites to egress and invade again. Two sets of magnetically purified schizonts (~40 to 42 hpi) were resuspended with fresh RBCs and incubated for 2 h in the presence of 10 μM inhibitors. In one set of cultures, the inhibitors were removed after 2 h by washing with RPMI and resuspending the cells in normal culture medium, while for the other set, the inhibitor was maintained for another 11 to 13 h (until 55 hpi). Following this, both sets of cultures were harvested and analyzed by flow cytometry. These experiments revealed remarkable differences among the molecules tested. For example, removal of drug pressure after initial exposure allowed parasites to egress normally in the case of MMV019127, MMV000642, MMV007181, MMV666061, MMV008956, and MMV665785. This was observed from the decreased presence of schizonts (>2-fold decrease) (Fig. 6C) and the corresponding increase in ring stage infection (>3-fold increase) (Fig. 6D) in washed cultures at 55 hpi. In contrast, the inhibition of the schizont-to-ring transition seen with MMV019266, MMV667490, and MMV665831 was not reversed when the inhibitors were washed away. The most significant reversal of the inhibitory effect was observed in the case of MMV007181, where inhibitor removal resulted in a >3-fold decrease and a >5-fold increase in schizont and ring populations, respectively. On the contrary, the least reversal of inhibition was seen in the case of MMV665831, reconfirming that this molecule is interfering primarily with egress and not invasion. Observation of egress inhibition even after the inhibitor is washed away could indicate that either the inhibitor has irreversibly modified its target or the target activity was inhibited at precisely the time when it was required for facilitation of egress (coinciding with the 2-h inhibitor incubation period between 40 and 44 hpi). Thus, these results indicate distinct modes of action and target selectivity for these compounds on *P. falciparum* development and maturation during late schizogony.

*P. falciparum* egress is known to be mediated by cascading effects of several proteases (56), and protease inhibitors are potent blockers of parasite egress. To test whether any of the Malaria Box molecules identified as egress inhibitors have broadly specific protease-inhibiting activity, we tested their effect on *P. falciparum* DV physiology, which requires coordinated action by many proteases working in concert. We reasoned that this would also allow us to test the specificity of the Malaria Box egress inhibitors. We adopted a previously reported method to identify *P. falciparum* DV physiology disrupters (59, 60), using the calcium reporter dye Fluo-4 AM to monitor alterations in DV permeability and staining with JC-1 probe to determine mitochondrial membrane integrity and cell viability. The results of this assay indicated that only two molecules, MMV006087 and MMV665879, affect DV integrity at 1 µM. None of the 26 egress inhibitors showed DV disruption activity. This was further verified through microscopic examination of trophozoites treated with the inhibitors at their estimated R_50_ values (Fig. S9).

10.1128/mSphere.00534-17.9 FIG S9  Demonstration of the lack of DV disintegration activity of egress inhibitors. Young trophozoite stage parasites (24 to 26 hpi, at 3% parasitemia) were treated with the 26 egress inhibitors at the respective R_50_ concentrations. Giemsa-stained thin smears prepared at 4 h postincubation confirmed that these molecules do not affect DV integrity. Parasites treated with 1% DMSO were used as negative controls (top left panel), while chloroquine- and E-64-treated parasites were used as positive controls (top right panel). Download FIG S9, PDF file, 0.2 MB. Copyright © 2018 Subramanian et al. 2018 Subramanian et al. This content is distributed under the terms of the Creative Commons Attribution 4.0 International license.

### Effects of *P. falciparum* egress inhibitors on *T. gondii* egress.

**FIG 7:**
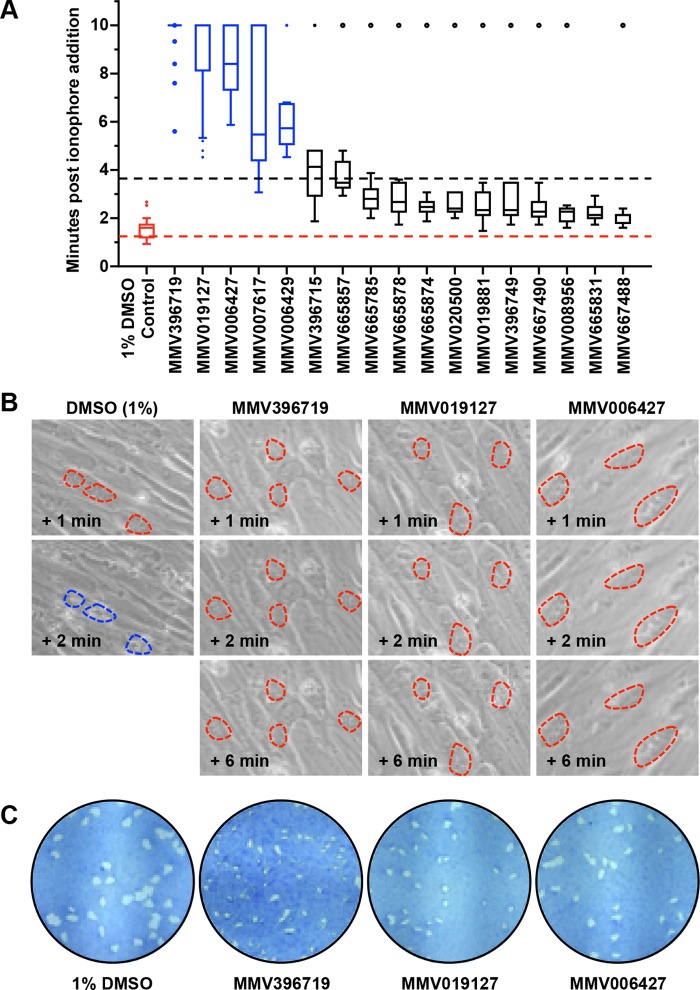
Inhibition of calcium ionophore-mediated egress of *T. gondii* tachyzoites. (A) Whisker plots of the timing of ionophore-induced egress of parasites following 24 h of treatment with selected Malaria Box molecules at 10 µM. Red, 1% DMSO-treated control cells; blue, inhibitors for which the median egress time was >4 min. (B) Time-lapse microscopic images showing ionophore-induced egress block following treatment with 10 µM inhibitor for 24 h. Red dashed lines mark intact vacuoles; blue dashed lines mark egressed vacuoles. (C) Formation of plaques with reduced size in the presence of the egress inhibitors indicated, in comparison with a 1% DMSO-treated control.

Host cell cytolysis pathways of *T. gondii* and *P. falciparum* are known to have conserved aspects (61–65). Therefore, we wanted to test if the Malaria Box molecules affecting the egress of *P. falciparum* are also capable of inhibiting the egress of *T. gondii* tachyzoites. The 26 molecules for which a detailed examination of *P. falciparum* egress inhibition was carried out were tested against *T. gondii* in the ionophore-induced egress assay. At 10 μM, 9 of the 26 molecules had a cytotoxic effect on HFF cells, which disqualified them from further *T. gondii* egress studies. Of the 17 remaining molecules, 5 (MMV396719, MMV019127, MMV007617, MMV006429, and MMV006427) were found to have a significant inhibitory effect on *T. gondii* egress. In particular, MMV396719 and MMV019127 were very potent. Even at 10 min after ionophore treatment, ≥80% of the *T. gondii* vacuoles remained intact (Fig. 7A and B). Notably, all of these molecules, except MMV019127, are known to disrupt sodium and pH homeostasis in *P. falciparum* (66).

The fact that none of these molecules were subjects of egress studies until now in the broader context of antiparasitic target discovery highlights the utility of comparative screening efforts such as those undertaken in this study. With the identification of five molecules in the potent and proven library compiled by MMV with comparable phenotypic effects on two parasites with distinct host cell preferences, an avenue to probe conserved targets that could be explored for biochemical and genetic validation in future work is established.

## DISCUSSION

The MMV Malaria Box molecules (4) have been extensively studied for their inhibitory potential against asexual and sexual stages of *P. falciparum* (24). This valuable antimalarial collection has also been screened against other parasitic species, such as kinetoplastids (19–21), helminths (17), *Babesia* (67), *Theileria* (68), *Cryptosporidium* (18), *Toxoplasma* (41), *Giardia* (69), and *Entamoeba* (41). These screens primarily focused on determining killing efficacies in whole-organism assays. However, little information is available on their mode of action in these parasites and only a small subset of molecules in this library have been successfully mapped to their targets.

Chemically induced phenotypes can facilitate downstream mechanistic studies, as they often serve as reliable indicators of the cellular pathways perturbed by the molecule of interest. This requires customized screening campaigns focused on the phenotype(s) of interest and is only possible when morphologically distinct cellular phenotypes can be linked to specific cellular pathways and targets. *P. falciparum* and *T. gondii*, two well-studied parasites, offer a good choice for carrying out such phenotypic screens, especially since experimental tools and reagents are readily available to dissect chemically induced cellular phenotypes. For instance, phenotypic features associated with impaired growth kinetics (i.e., fast versus delayed killing), host cytolysis (the endpoint of an intracellular replicative cycle of the parasite), and host invasion (to establish a new infectious cycle) are well characterized in both of these parasites. Furthermore, because of their shared evolutionary history, orthologous proteins in *P. falciparum* and *T. gondii* are often associated with similar cellular processes and, importantly, are likely to share sensitivity to inhibitors that affect these unique life stage events. This reasoning motivated us to undertake targeted phenotypic screening of Malaria Box molecules to identify delayed death, egress, and invasion inhibitors in *T. gondii* and *P. falciparum*, respectively. Establishing the linkage between unique chemical scaffolds and their resultant cellular phenotypes on two evolutionarily related yet distinct parasites will provide an avenue for conducting detailed mechanistic studies with the organism of choice by biochemical, cell biological, and/or genetic approaches, as appropriate.

In this study, we first determined the EC_50_s of the 390 Malaria Box molecules procured from the MMV for *P. falciparum* and *T. gondii* to confirm that the activities of the molecules obtained are in agreement with previously published data (24). For *P. falciparum*, we found that 168 molecules had nanomolar EC_50_s and 99 molecules had EC_50_s between 1 and 3 µM, indicating overall agreement with the data previously released by MMV (Table S1). However, in the case of *T. gondii*, we obtained results markedly different from previously published work (41). The reported study used a short-term (24-h) killing assay performed with *T. gondii* tachyzoites and identified only seven molecules with significant antitoxoplasma activity, of which only one, MMV007791, was reported to have nanomolar activity. In our assays, parasites were incubated for 48 h with the Malaria Box molecules before they were checked for growth inhibition. We used a transgenic strain of *T. gondii* expressing the luciferase gene as a reporter, where the luminescence readout is more sensitive and reproducible than the previously used method of monitoring parasite killing. Overall, we found that 199 Malaria Box molecules exhibited >50% growth inhibition and 119 had a >80% growth inhibition effect on *T. gondii* tachyzoites at a 10 µM concentration. Of the 49 molecules that exhibited nanomolar EC_50_s against *T. gondii*, 24 had nanomolar potency against *P. falciparum* as well (Fig. 2; Table S1). Not much is known from the available literature regarding the mechanism of action of these 24 molecules. Of these molecules, a few, MMV665941, MMV006389, and MMV665977, were reported to inhibit *P. falciparum* gametocyte development (10) and MMV665941 was found to have transmission-blocking activity (70). In a separate study, MMV665886 was found to have translation inhibition activity against *Plasmodium* (71). Metabolomic profiling has revealed that MMV666596, MMV665977, MMV006309, and MMV665940 induce metabolic changes consistent with inhibition of pyrimidine biosynthesis, while MMV396669 and MMV000963 induce metabolic changes consistent with inhibition of hemoglobin metabolism (25, 26). Further mechanistic studies of these potent molecules, especially in *T. gondii*, have yet to be performed.

Next, among the molecules that showed <20% growth inhibition in *T. gondii* killing assays, we identified 30 that completely inhibited plaque formation, suggesting that they cause delayed death of the parasite. Notably, about half of these molecules have potent and immediate-killing antiplasmodial activity, indicating that these molecules may be acting on distinct targets in *T. gondii* and *P. falciparum*. The mechanism of delayed killing of apicomplexan parasites has been extensively studied and is linked to the inhibition of apicoplast housekeeping functions (43, 44). Examples of delayed-death inhibitors include antibiotics such as azithromycin, clindamycin, and doxycycline, which target the 70S ribosome, and ciprofloxacin, which targets the apicoplast-associated DNA gyrase (42). These antibiotics are equally effective in causing delayed death in *P. falciparum* and *T. gondii*, although phenotypic differences exist (43).

We identified four molecules, MMV008455, MMV020885, MMV019199, and MMV666109, that induced apicoplast missegregation during daughter cell formation in *T. gondii*. A previous attempt to identify Malaria Box delayed-death inhibitors of *P. falciparum* identified MMV008138, which was found to target the 2-C-methyl-d-erythritol 4-phosphate cytidylyltransferase (IspD) enzyme required for isoprenoid biosynthesis in the apicoplast (72). MMV008138, however, exhibits no immediate-killing or delayed-death effects on *T. gondii*. It is worth noting that although *P. falciparum* undergoes delayed death, its association with a defective plastid segregation phenotype can vary markedly (72, 73). The four MMV Malaria Box molecules that target the apicoplast are chemically distinct from the macrolide, tetracycline, lincosamide, and fluoroquinolone class of compounds (Fig. 4B) that are known to cause plastid missegregation and delayed death in *T. gondii*. Thus, it will be interesting to study the mechanism by which these molecules cause delayed death in *T. gondii*. For the remaining 26 molecules, which cause delayed death but have no apparent phenotypic effect on the plastids in our assays, it needs to be investigated whether they act by targeting plastid-associated housekeeping functions.

Parasite release is accompanied by host cell cytolysis, involving the concerted action of pore-forming proteins, kinases, proteases, and osmotic factors to compromise the host membrane and trigger catabolic enzymes to eventually dismantle the infected cell (56). In the case of *P. falciparum*, molecules targeting the cysteine and serine type proteases have differential inhibitory effects on the host RBC membrane and PV membrane, respectively (39, 74), resulting in readily distinguishable microscopic features. Some of these effects are phenocopied in *T. gondii* as well (38, 75). We identified 26 molecules from the Malaria Box collection that inhibited the egress of *P. falciparum* merozoites from iRBCs with low micromolar to nanomolar potency. Many of these molecules were found to have a comparable inhibitory effect on drug-resistant strains of *P. falciparum* as well (Fig. 5B). Of the 26 molecules tested here, 10 (MMV000653, MMV000642, MMV007617, MMV396715, MMV396719 MMV006429, MMV396749, MMV006427, MMV665878, and MMV666025) have been previously shown to affect parasite Na^+^ and pH homeostasis in a manner similar to that of the *Pf*ATP4 inhibitor (66) spiroindolone KAE609 (also known as NITD609) (76, 77). While it is possible for these molecules to target *Pf*ATP4 and thereby indirectly affect the egress-related processes, the likelihood of their acting via novel targets and pathways cannot be ruled out. Intriguingly, MMV396749, a benzimidazole, has significant structural similarity to spiroindolone KAE609 and was reported to inhibit the liver stage development of malaria parasites (78). In our egress/invasion assays, MMV000642, MMV396715, MMV396719, MMV006429, MMV665878, and MMV665831 showed >50% inhibition of ring formation at 0.3 µM. Microscopic analysis revealed three mechanistically distinct classes of molecules, RBC membrane rupture blockers, PV rupture blockers, and invasion blockers (Fig. 6). By washing away the drug, we were able to reverse the inhibitory effect of selected molecules. This is interesting, as it indicates that the timing of inhibition with respect to the egress phenomenon is critical for effective inhibition. This is also in agreement with previous findings showing that critical factors facilitating parasite egress from host cells, such as proteases, are present in their active forms only for a short duration when egress is in process (39, 58). Moreover, the *Plasmodium* egress/invasion inhibitors did not affect the physiology of the DV, ruling out the possibility that they are broadly specific protease inhibitors.

It has been shown that sequential steps during invasion, such as host recognition, binding, local proteolysis, or junction formation, can be manipulated using small molecules (or antibodies), generating phenotypes that can be indicative of the specific step being affected. Among the 26 compounds that affected the transition of *P. falciparum* schizonts to rings, we were able to identify 2 (MMV006429 and MMV665878) that specifically affected invasion (Fig. 6B). Most importantly, we found that five Malaria Box molecules that were identified as potent blockers of *P. falciparum* egress were also highly effective in blocking the egress of calcium ionophore-induced *T. gondii* tachyzoites. Two of these molecules, MMV396719 and MMV019127, that strongly inhibit parasite egress are potential candidates for further mechanistic studies.

Taken together, our results provide comprehensive documentation of selective phenotypic effects of MMV Malaria Box molecules against *P. falciparum* and *T. gondii*. By employing complementary techniques, we have prioritized at least a dozen inhibitors that selectively impair (i) apicoplast segregation, (ii) host cell invasion, and/or (iii) parasite egress. A majority of these hits are “drug-like” molecules with not only proven antimalarial efficacy but also chemical characteristics that make them amenable to subsequent medicinal chemistry work. Further investigations dissecting their mode of action by combining biochemical and genetic means would allow the community to exploit these molecules for therapeutic use against malaria and toxoplasmosis.

## MATERIALS AND METHODS

### Ethics statement.

All experimental procedures were conducted in accordance with approved guidelines from the respective institutions. The blood used to culture malaria parasites was purchased from the Interstate Blood Bank in the United States (for work conducted in Singapore) or the Poona Serological Trust Blood Bank in Pune, India, and was collected from anonymous donors.

### Blood collection and storage.

Blood collected in EDTA tubes (VACUETTE EDTA Tubes; Greiner Bio-One) was washed thrice with RPMI 1640 (Sigma-Aldrich) to remove the buffy coat. Washed RBCs were stored for a maximum of 4 weeks at 4°C in complete malaria culture medium (see below) at 50% hematocrit and washed once prior to every use.

### Preparation of stock and working concentrations of drugs.

The Malaria Box libraries received from MMV, Geneva, Switzerland (two separate shipments to the collaborating labs in Singapore and India), contained 390 compounds as 10 mM stock solutions in a 96-well plate format and were stored in −80°C until use. Ninety microliters of cell culture grade DMSO (Sigma-Aldrich, United States) was added to 10 mM stocks to make 1 mM working stocks that were eventually used to set up the various assays. For both *P. falciparum* and *T. gondii*, the top concentration used in either killing or phenotype assays was 10 µM. For EC_50_ determination, 2-fold serial dilutions of the compounds were made by starting at 10 µM. Atovaquone (*P. falciparum* and *T. gondii*), chloroquine (*P. falciparum*), clindamycin (*T. gondii*), and chloramphenicol (*T. gondii*) were used as standard positive controls in the killing and phenotypic assays. The concentrations of the standard drugs used in various assays are given in the relevant sections.

### *P. falciparum* culture protocols.

*P. falciparum* (strain 3D7) was used for all experiments, unless stated otherwise. Chloroquine-resistant (K1) and artemisinin-resistant (KH004-003) parasites were obtained from MR4 and Peter Preiser’s lab, Nanyang Technological University, Singapore, respectively. Parasites were cultured at 2.5% hematocrit in RPMI-HEPES medium at pH 7.4 supplemented with hypoxanthine at 50 μg·ml^−1^, NaHCO_3_ at 25 mM, gentamicin at 2.5 μg·ml^−1^, and AlbuMAX II (Gibco) at 0.5% (wt/vol). Late-stage parasites were enriched with a MACS device (Miltenyi Biotech, Bergisch Gladbach, Germany) as reported elsewhere (79). To obtain enriched ring stage parasites, a standard sorbitol synchronization method was used.

### *T. gondii* culture protocols.

Tachyzoite stage *T. gondii* (type I RH strain) parasites were routinely maintained in HFF (ATCC) monolayers by standard protocols (80). Briefly, HFF cells were grown in Dulbecco’s modified Eagle’s medium supplemented with 10% heat-inactivated fetal bovine serum (FBS; U.S. origin, Gibco), 25 mM HEPES, 2 mM GlutaMAX, and gentamicin at 50 µg/ml in a 37°C humidified incubator maintaining 5% CO_2_. Confluent monolayers of HFF cells were infected with 10^5^ freshly isolated, host cell-free *T. gondii* tachyzoites. The culture medium used for parasite growth was the same as that used for HFF cell maintenance, except that FBS was used at 1%. To isolate parasites residing within host cells, the infected HFF monolayer was scraped and passed through a 25-gauge needle to lyse the host cells and release the tachyzoites. The parasites were then separated from HFF cell debris by filtration through a membrane with a 3-μm pore size. Ten microliters of the 1:10-diluted filtrate was placed in a hemocytometer, and cell counts were obtained with the Countess machine (Thermo).

### Determination of EC_50_s for *P. falciparum.*

The assays used to determine EC_50_s for *P. falciparum* were carried out in a 96-well plate format by SYBR green staining-based parasitemia determination as previously reported (81, 82). The plates were first seeded with complete RPMI medium containing each MMV Malaria Box molecule at the required concentrations of 10 to 0.01 µM, followed by the addition of an iRBC suspension. The final hematocrit and parasitemia were maintained at 2.5 and ~2%, respectively. Each 96-well plate also included a standard antimalarial compound as a positive control (usually atovaquone or chloroquine at 1 µM) and 1% DMSO as a negative control. Inhibitor treatment was done in duplicate, while controls were set up as four replicates. Assay plates were incubated for 60 h under optimal growth conditions, followed by staining with 25 µl of 10× SYBR Green staining solution consisting of 0.5% Triton X-100 and 10× SYBR green I dye (Invitrogen) in phosphate-buffered saline at pH 7.4. Fluorescence readings were obtained after 15 min of incubation with a GloMax plate reader (Promega). The raw fluorescence readings were then processed using Microsoft Excel spreadsheets for dose response calculation and EC_50_ estimation.

### Determination of *T. gondii* growth inhibition and EC_50_s.

*T. gondii* RH strain tachyzoites expressing the firefly luciferase gene were used to identify Malaria Box molecules with antitoxoplasma activity and determine their EC_50_s. Details regarding the generation of transgenic parasites expressing luciferase and standardization of the luminescence assay are provided in the supplemental material. The assays were set up in a 96-well plate format, and 100 µl of culture medium containing 5 × 10^3^ parasites was inoculated into each well containing a confluent monolayer of HFF cells, preseeded with 100 µl of culture medium containing Malaria Box compounds either at 10 µM (for growth inhibition studies) or in serial 2-fold dilutions ranging from 10 to 0.01 µM (for EC_50_ determination). Each plate also included a standard drug as a positive control (usually atovaquone at 1 µM) and 1% DMSO as a negative control. Inhibitor treatment was done in triplicate for growth inhibition and in duplicate for EC_50 _determination. The controls were set up as four replicates each. After 48 h of growth under optimal conditions, 150 µl of culture medium was removed and 50 µl of 2× luciferase assay reagent (Promega) was added and mixed well. The plates are immediately read with a VarioScan plate reader (Thermo Fisher, United States). The raw luminescence readings were then processed by using Microsoft Excel spreadsheets for calculation of percent growth inhibition and EC_50_ estimation.

### *T. gondii* plaque formation and delayed-death assays.

The plaque-forming ability of *T. gondii* (RH strain) tachyzoites was tested to detect a delayed-death effect in Malaria Box molecules having <20% growth inhibition of tachyzoite stage *T. gondii* at a 10 µM inhibitor concentration in 48-h killing assays. The plaques assays were initiated by inoculating 50 tachyzoites into each well of a six-well plate containing confluent monolayers of HFF cells preseeded with selected Malaria Box compounds at 10 µM. The plates were left undisturbed for 8 to 10 days under optimal growth conditions, after which the infected monolayers were fixed with methanol and stained with crystal violet to visualize plaque formation. The plaques were then imaged, and all images were processed with ImageJ software to determine the plaque area and count in each well. The plaque area of inhibitor-treated cultures was compared with that of untreated and 1% DMSO-treated controls. Clindamycin (known to cause delayed death of *T. gondii*) at 10 µM and pyrimethamine or atovaquone at 1 µM were used as positive controls for parasite killing.

### Assay of apicoplast missegregation in *T. gondii.*

Transgenic RH-*Tgtpi-II-yfp T. gondii* parasites, in which the endogenous triosephosphate isomerase II gene is tagged with YFP, were used to track the apicoplast phenotype by microscopy. First, tachyzoite stage parasites (5 × 10^3^) were allowed to invade a confluent monolayer of HFF cells (first vacuole) in a 96-well plate in the presence of selected Malaria Box molecules (10 µM) identified as having a delayed-death effect on the parasite. After 48 h of incubation under optimal growth conditions, parasites were harvested by trypsin treatment, followed by passage of the infected cells by syringe through a 25-gauge needle to release free extracellular parasites. These parasites were then added to a fresh monolayer of HFF cells grown on coverslips in 24-well plates to initiate a second round of invasion (second vacuole), again in the presence of a 10 µM inhibitor concentration. After ~20 h of growth under optimal conditions, the coverslips were fixed with 3.5% paraformaldehyde, stained with 4',6-diamidino-2-phenylindole (DAPI) to visualize cell nuclei, and mounted on a glass slide with Fluoromount (Sigma). The coverslips were then imaged with a 63× objective fitted to an inverted fluorescence microscope (Carl Zeiss, Inc.). Apicoplast-associated YFP and nuclear DAPI were imaged with excitation/emission wavelength filter combinations of 514/527 nm and 350/470 nm, respectively.

### Calcium ionophore-induced egress of intracellular *T. gondii* tachyzoites.

The calcium ionophore A23187 was used to induce the egress of intracellular tachyzoites as previously reported (83). Briefly, at 24 hpi, when the vacuoles contained between 8 and 16 parasites, the plates were removed from the incubator and allowed to equilibrate to room temperature for 5 min before appropriate vacuoles were located for imaging with a 40× objective fitted to an inverted bright-field microscope (Primo Vert; Zeiss). After ionophore addition (final concentration, 1 µM) to the culture, the vacuoles were imaged continuously for 10 min. The timing of parasite egress following ionophore addition was monitored.

### Flow cytometry analysis of *P. falciparum* cultures.

Flow cytometry analysis was carried out to quantify parasitemia and determine the fractions of parasites in the ring and schizont stages. A benchtop flow cytometer (Accuri C6; BD Biosciences) was used, and at least 100,000 events were recorded in each sample. To determine parasitemia, a 50-μl culture aliquot was fixed with phosphate-buffered saline (PBS) containing 0.1% glutaraldehyde (Sigma-Aldrich) at 4°C overnight. Cells were then washed in PBS, permeabilized with PBS containing 0.25% Triton X-100 (Sigma-Aldrich) for 10 min at room temperature, and finally washed again in PBS. After being washed, samples were incubated with Hoechst 33342 (Thermo Fisher) at 25 μg/ml for 30 min in the dark and counted by flow cytometry as previously reported (50). Data analysis and statistical calculations were performed with GraphPad Prism (GraphPad Software, Inc.) in accordance with the recommended protocol for nonlinear regression of a log(inhibitor)-versus-response curve.

### Microscopic examination of *P. falciparum.*

Thin smears of *P. falciparum* cultures were prepared on glass slides, fixed with 100% methanol, and stained with fresh Giemsa (Sigma) solution made in filtered distilled water. Smears were examined with a 100× oil immersion objective and a standard phase-contrast microscope (Leica). Images from the smears were captured with an Olympus digital camera and processed with Adobe Photoshop.

### Monitoring of the *P. falciparum* egress phenotype and determination of R_50_ values.

Tightly synchronized schizont stage parasites (~40 to 42 hpi) at 1% parasitemia and 2.5% hematocrit were incubated with selected drugs at 10, 3, 1, 0.3, and 0.1 µM for 12 h and harvested for flow cytometry analysis. At the time of analysis, in DMSO-treated controls, schizont maturation and ring stage formation were almost 100%. Heparin (invasion inhibitor)-treated parasites served as negative controls for egress inhibition, while E-64 (egress inhibitor) was included as a positive control for egress inhibition. In parallel, Giemsa-stained smears were made at 52 hpi to monitor and confirm the rupture phenotypes. For a more detailed analysis of egress and/or invasion inhibition by the compounds, the above protocol was used, except with a higher parasitemia level of 2.5%.

### Molecular protocols.

The transgenic *T. gondii* parasite (RH) strains used in this study are RH-Luc and RH-*Tgtpi-II-yfp*. RH-Luc, which constitutively expresses the firefly luciferase gene, was used for killing assays. The firefly luciferase gene (PCR amplified from plasmid pGL3 [Promega]) was cloned into a modified pBluescript plasmid backbone as a BglII and NheI fragment for expression under the constitutively active *T. gondii* β-tubulin 5′ untranslated region (UTR) and the *Tg*SAG 3′ UTR. This plasmid includes the dihydrofolate reductase (DHFR) expression cassette for the selection of stable lines of transfected parasites with pyrimethamine.

RH-*Tgtpi-II-yfp*, which constitutively expresses the triose phosphate isomerase II gene tagged with YFP, was used for apicoplast missegregation studies. Since the TPI-II protein is naturally targeted to the apicoplast, in this transgenic parasite, the organelle is marked by YFP fluorescence. The genomic locus tagging plasmid construct used to generate transgenic parasites expressing RH-*Tgtpi-II-yfp* was made as follows. Genomic DNA was isolated from tachyzoite stage *T. gondii* with a commercial kit (Qiagen) in accordance with the manufacturer’s protocol. The Topo 2.1 cloning vector (Invitrogen) was modified to make a 3′ YFP tagging plasmid. PCR-amplified 1.7-kb *T. gondii* genomic DNA corresponding to the 3′ region of the *Tgtpi-II* gene locus [TGME49_233500 and TGME49_chrVIII, 2,682,525 and 2,688,704 (−)], which includes the codon for the last amino acid but excludes the stop codon, was cloned as a HindIII and AvrII fragment. The YFP coding sequence, along with the *Tgdhfr* 3′ UTR region, was then cloned downstream of the *Tgtpi-II* genomic region. A DHFR selection cassette, used to obtain a stable line by pyrimethamine selection, was cloned into the tagging plasmid as a NotI fragment. Prior to parasite transfection, the tagging plasmid was linearized with the BstXI enzyme, which cuts in the middle of the 1.7-kb *Tgtpi-II* genomic fragment (Fig. S4).

### Generation of *T. gondii* transgenic lines.

Freshly isolated *T. gondii* tachyzoites were washed and resuspended in complete parasite culture medium. A 50-µg sample of linearized plasmid DNA dissolved in 100 µl of medium was mixed with 300 µl of a parasite suspension (~1 × 10^7^ cells), and the mixture was electroporated with a Gene Pulser electroporation system (Bio-Rad) with capacitance and voltage settings of 10 μF and 1.5 kV, respectively. Transfected parasites were then allowed to infect a fresh layer of HFF cells and incubated for 12 h under optimal growth conditions. Infected HFF monolayers were then washed once, and 10 µM pyrimethamine was added to the culture medium and incubated for 48 h to obtain stable transgenic lines, which were then cloned by the limiting-dilution method. A clonal isolate of the firefly luciferase-expressing parasite was tested for linearity in luminescence detection over a 2-fold dilution series of parasite numbers. The results (Fig. S1) indicate good linearity between parasite counts and luminescence readouts in the range of 10^4^ to 10^8^ parasites. We tested the linearity of the luminescence detection assay after inoculating HFF monolayers into 96-well plates with various numbers of parasites and allowing them to proliferate for 48 h under standard growth conditions. On the basis of the results obtained from this experiment (Fig. S1B), we decided to inoculate 5 × 10^3^ tachyzoite stage parasites per well for the drug inhibition assays.
