# Erratum for Subramanian et al., “Targeted Phenotypic Screening in *Plasmodium falciparum* and *Toxoplasma gondii* Reveals Novel Modes of Action of Medicines for Malaria Venture Malaria Box Molecules”

**DOI:** 10.1128/mSphere.00159-19

**Published:** 2019-03-13

**Authors:** Gowtham Subramanian, Meenakshi A. Belekar, Anurag Shukla, Jie Xin Tong, Ameya Sinha, Trang T. T. Chu, Akshay S. Kulkarni, Peter R. Preiser, D. Srinivasa Reddy, Kevin S. W. Tan, Dhanasekaran Shanmugam, Rajesh Chandramohanadas

**Affiliations:** a Pillar of Engineering Product Development, Singapore University of Technology and Design, Singapore; b Biochemical Sciences Division, CSIR National Chemical Laboratory, Pune, India; c Academy of Scientific and Innovative Research, Ghaziabad, India; d Department of Microbiology and Immunology, National University of Singapore, Singapore; e Organic Chemistry Division, CSIR National Chemical Laboratory, Pune, India; f School of the Biological Sciences, Nanyang Technological University, Singapore

## ERRATUM

Volume 3, no. 1, e00534-17, 2018, https://doi.org/10.1128/mSphere.00534-17. One institution was missing from the list of author affiliations in the original paper. The list of institutional affiliations should appear as given in this erratum.

