# Supplementary figures and images for: Targeted Phenotypic Screening in Plasmodium falciparum and Toxoplasma gondii Reveals Novel Modes of Action of Medicines for Malaria Venture Malaria Box Molecules
Source: mSphere. 2018 Jan 24;3(1):e00534-17. doi: 10.1128/mSphere.00534-17 (PMC5770543; doi:10.1128/mSphere.00534-17)

# Supplementary Figure-S1

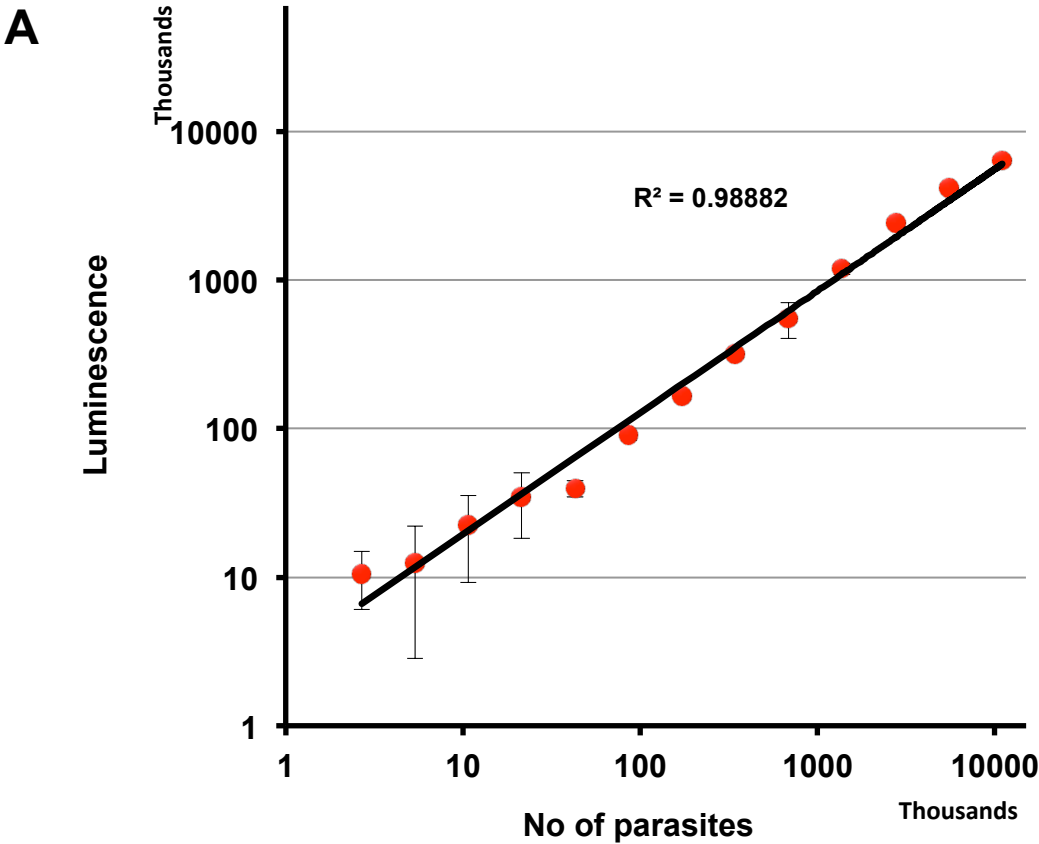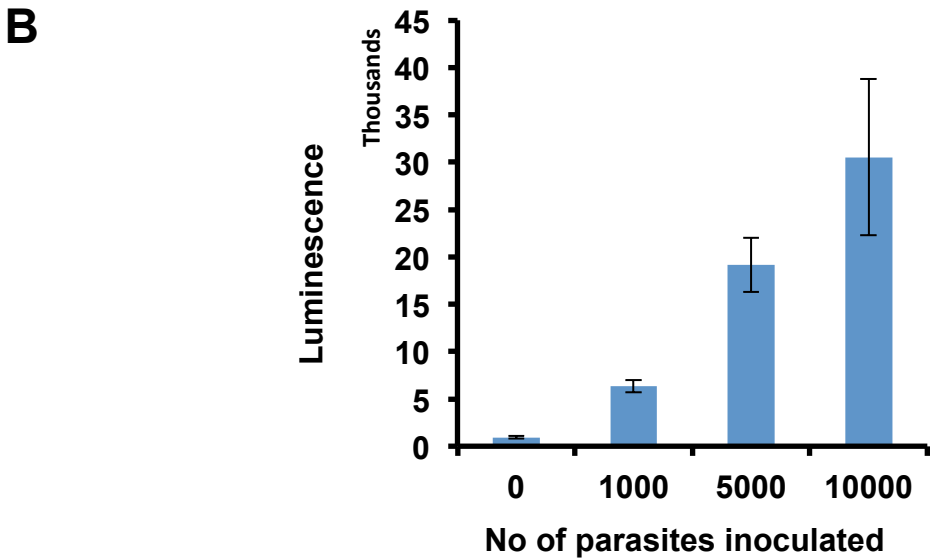

Supplement: FIG S1 [file sph001182457sf1.pdf]

**Supplementary Figure-S2**

**A**

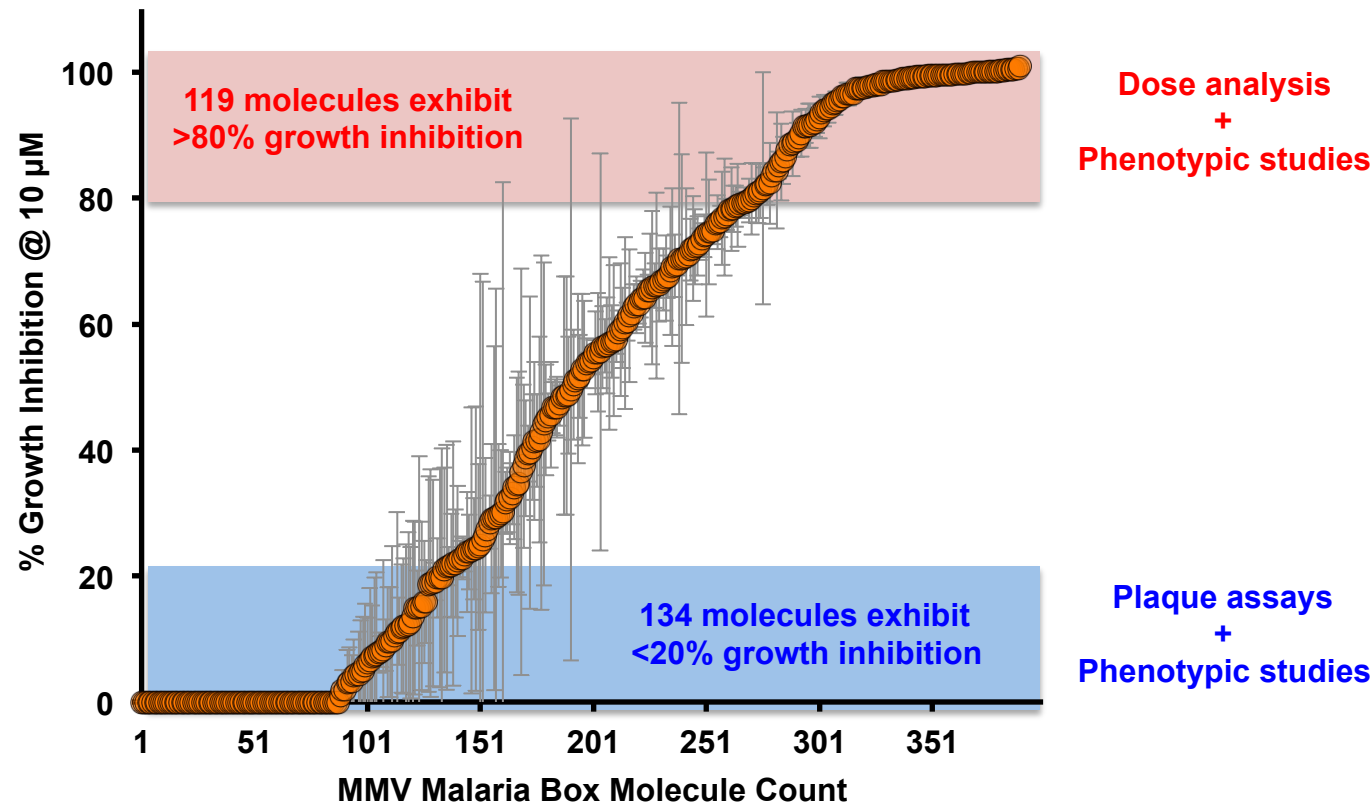

**B**

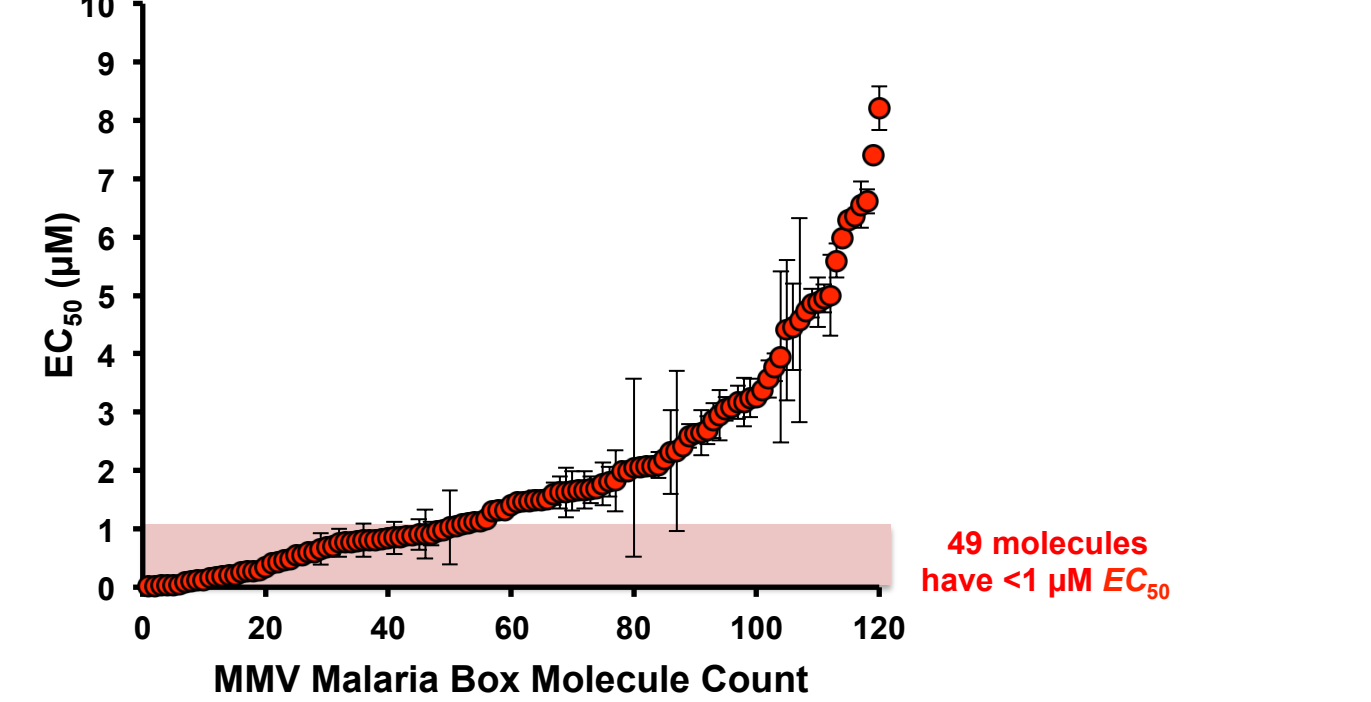

Supplement: FIG S2 [file sph001182457sf2.pdf]

# Supplementary Figure-S3

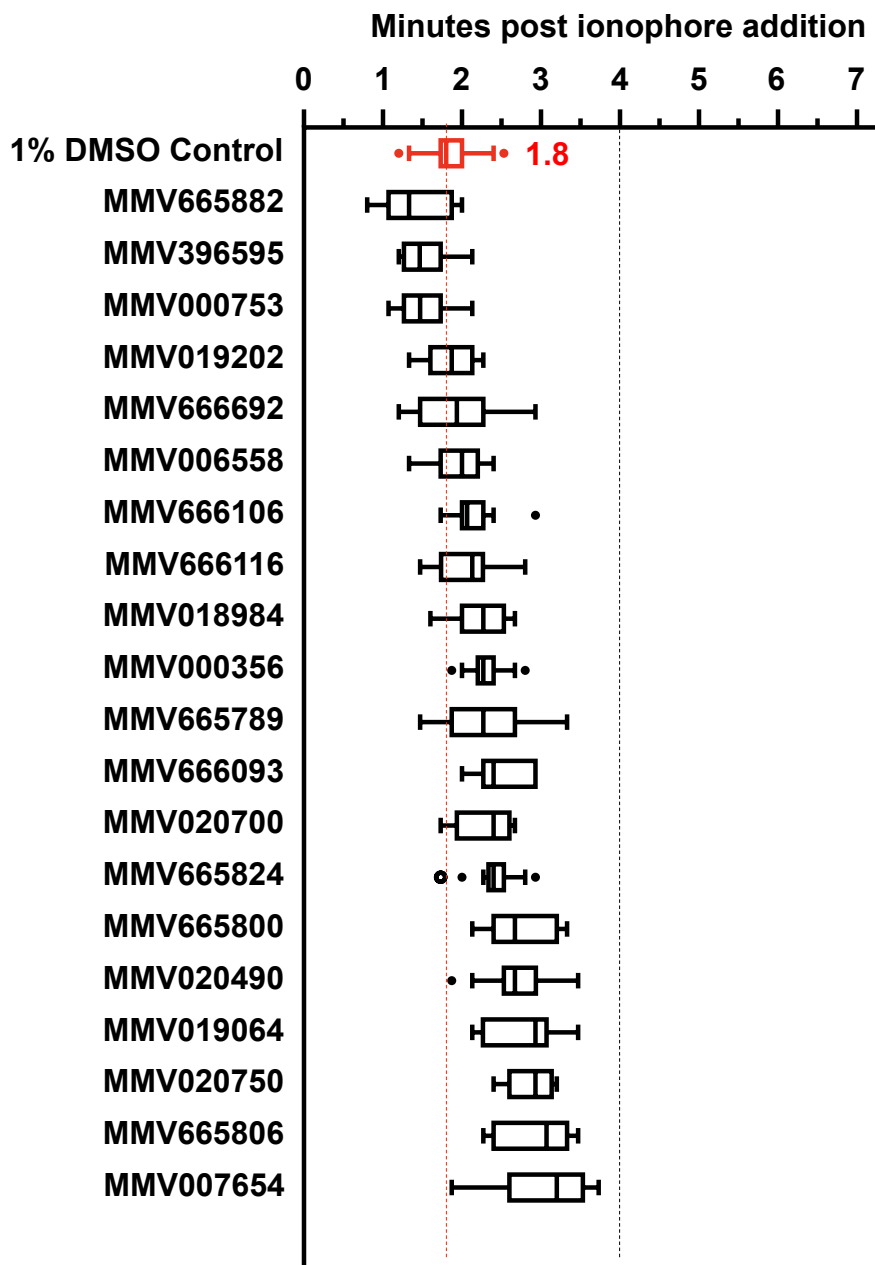

Supplement: FIG S3 [file sph001182457sf3.pdf]

# Supplementary Figure-S4

A

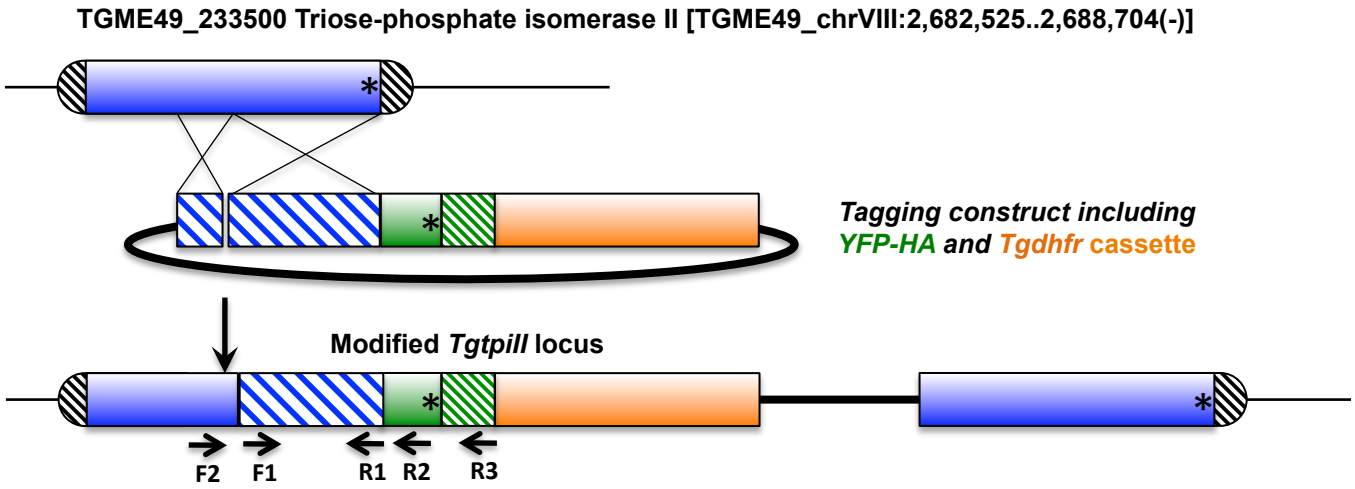

B

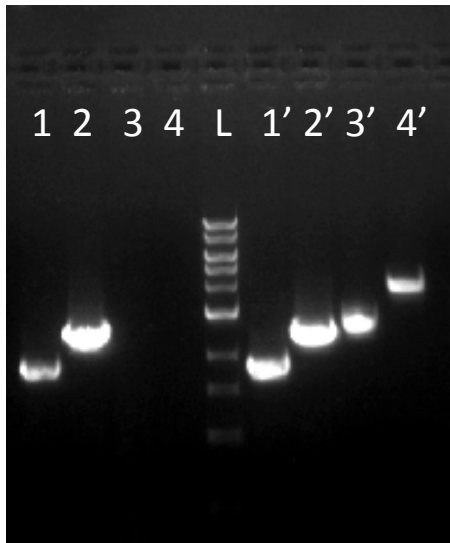

1,1'-F1,R1 (1.7kb)  
2,2'-F2,R1 (2.4kb)  
3,3'-F2,R2 (2.7kb)  
4,4'-F2,R3 (3.9kb)

RH Wt  
(Parental strain)

RH *Tgtpill*-  
YFP

Supplement: FIG S4 [file sph001182457sf4.pdf]

# Supplementary Figure-S5

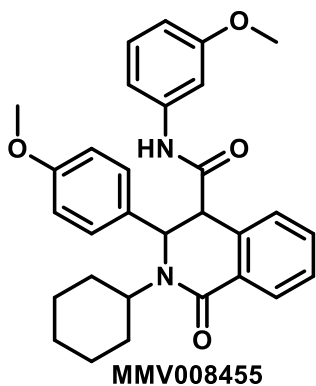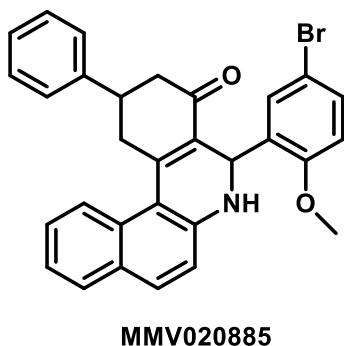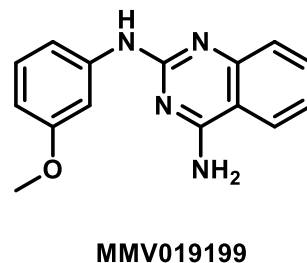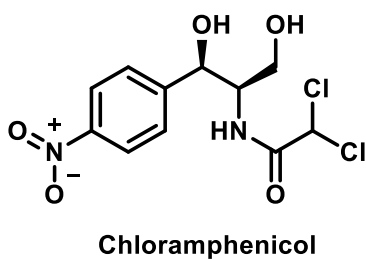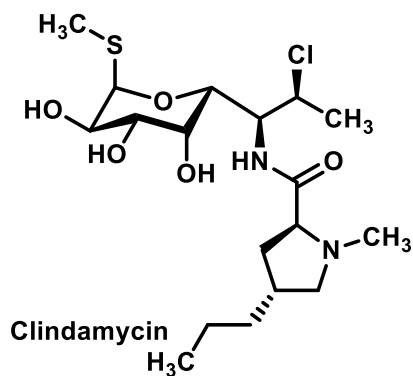

Supplement: FIG S5 [file sph001182457sf5.pdf]
