# Supplementary material for: Targeted Phenotypic Screening in Plasmodium falciparum and Toxoplasma gondii Reveals Novel Modes of Action of Medicines for Malaria Venture Malaria Box Molecules
Source: mSphere. 2018 Jan 24;3(1):e00534-17. doi: 10.1128/mSphere.00534-17 (PMC5770543; doi:10.1128/mSphere.00534-17)

Supplementary Figure-S6

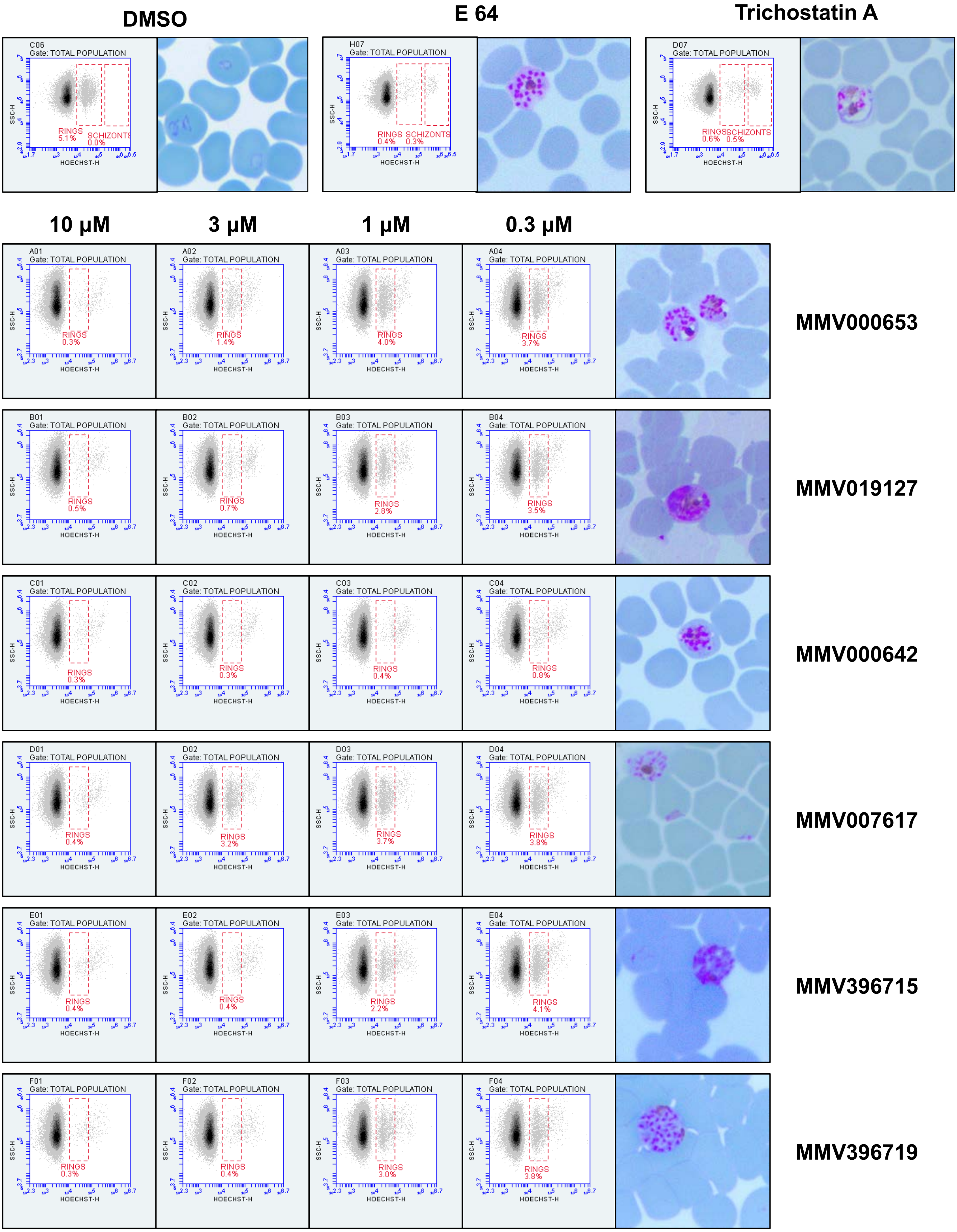

Supplementary Figure-S6 continued

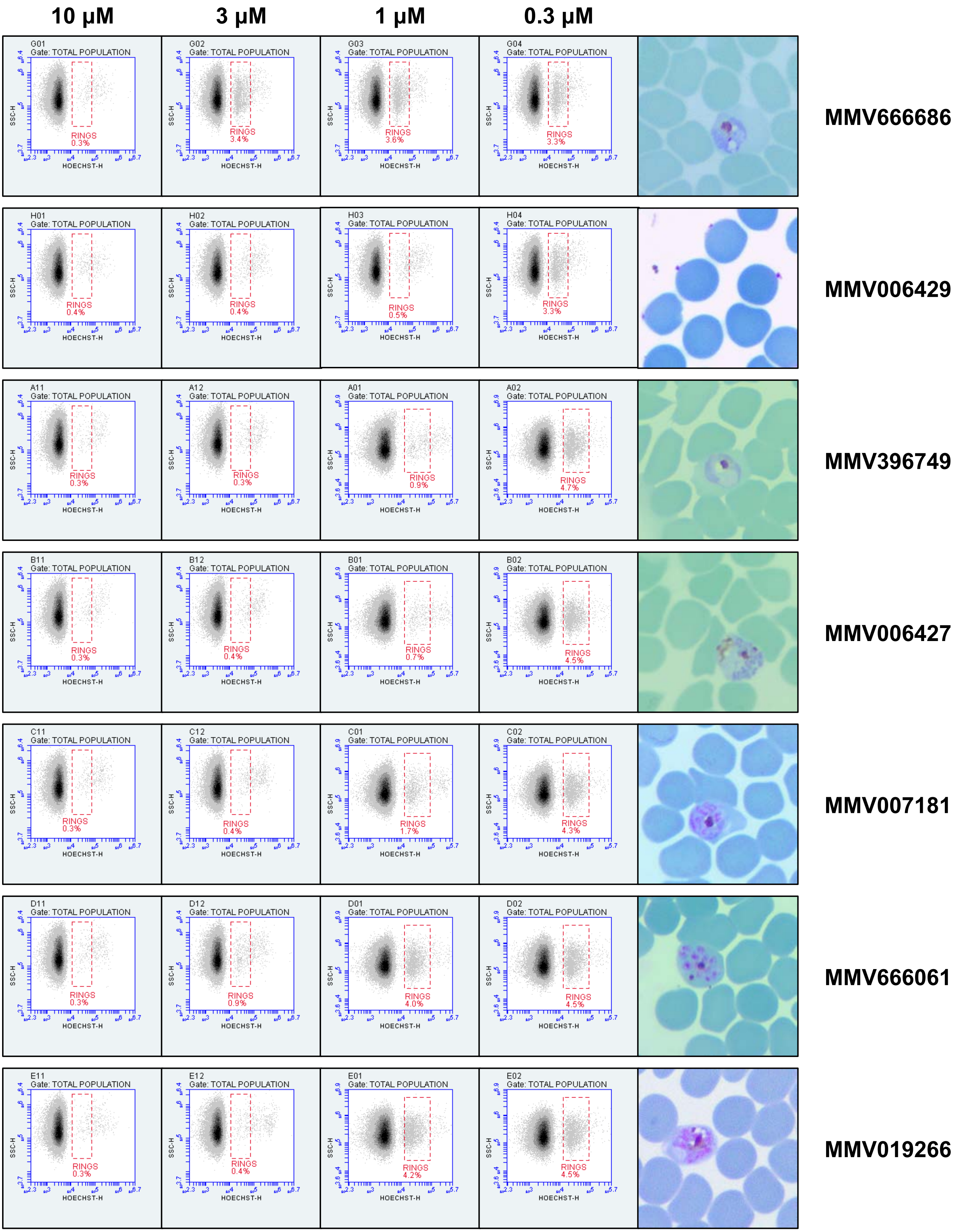

Supplementary Figure-S6 continued

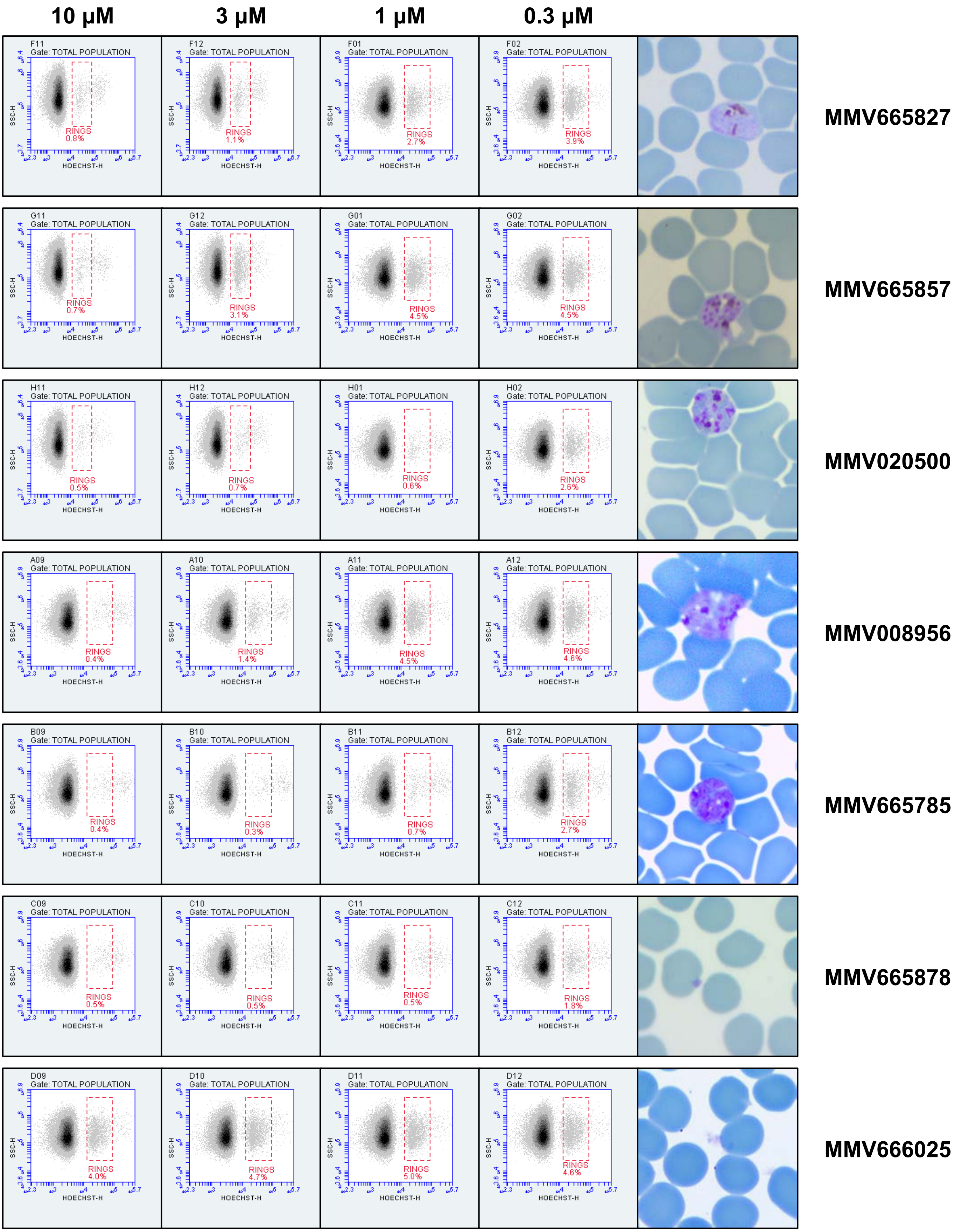

Supplementary Figure-S6 continued

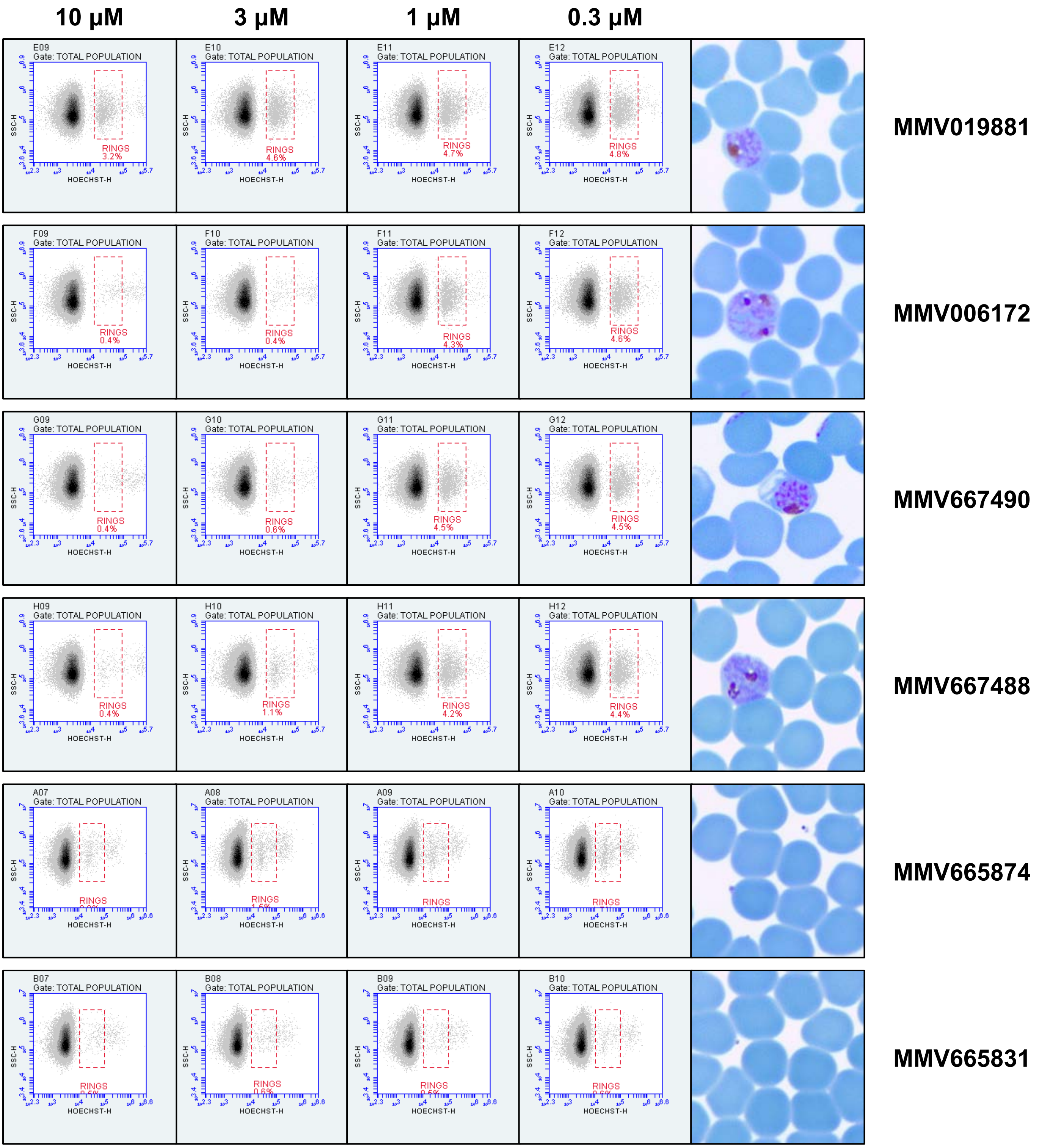

Supplementary Figure-S6 continued

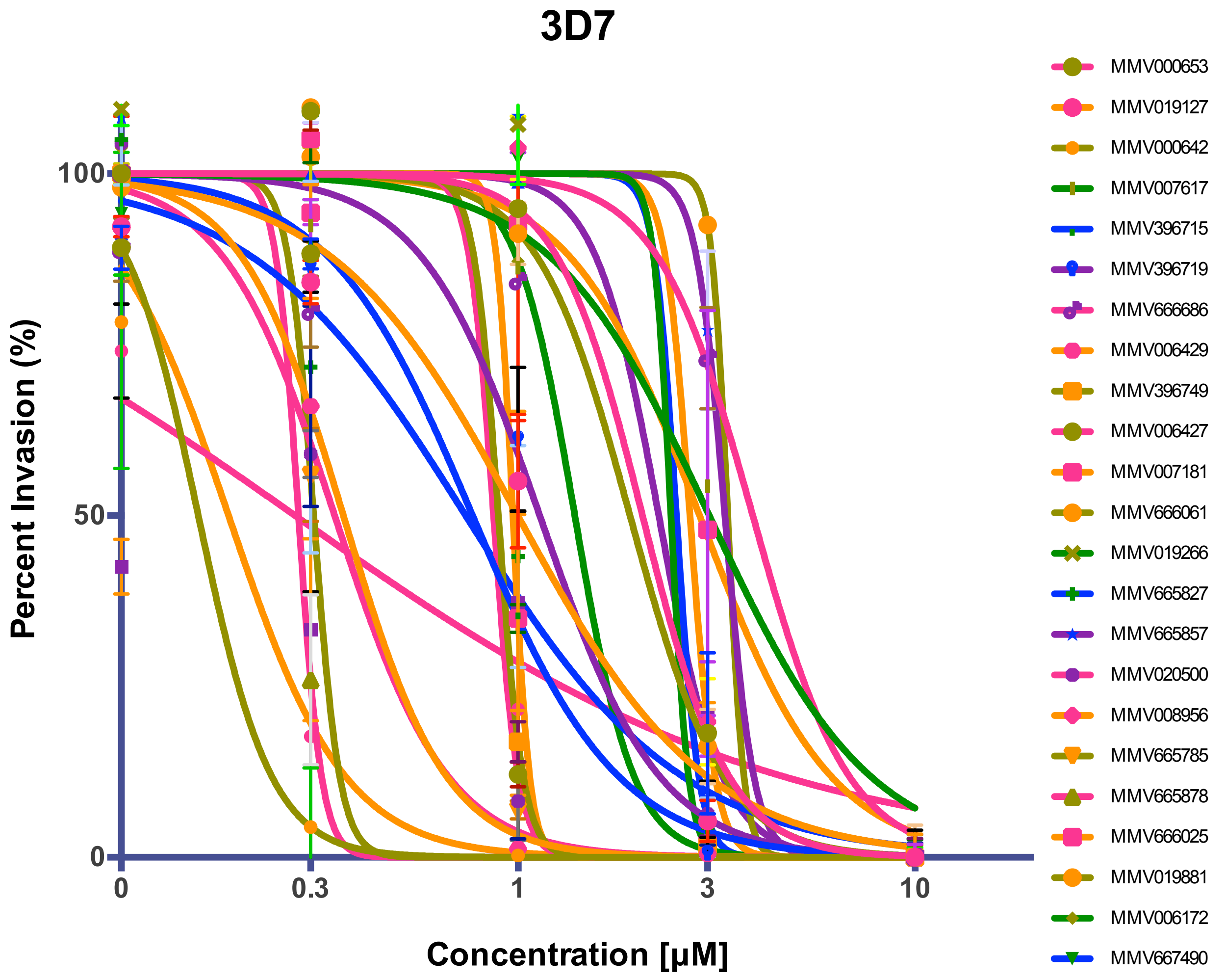

Supplement: FIG S6 [file sph001182457sf6.pdf]
