# Supplementary material for: Targeted Phenotypic Screening in Plasmodium falciparum and Toxoplasma gondii Reveals Novel Modes of Action of Medicines for Malaria Venture Malaria Box Molecules
Source: mSphere. 2018 Jan 24;3(1):e00534-17. doi: 10.1128/mSphere.00534-17 (PMC5770543; doi:10.1128/mSphere.00534-17)

Supplementary Figure-S7

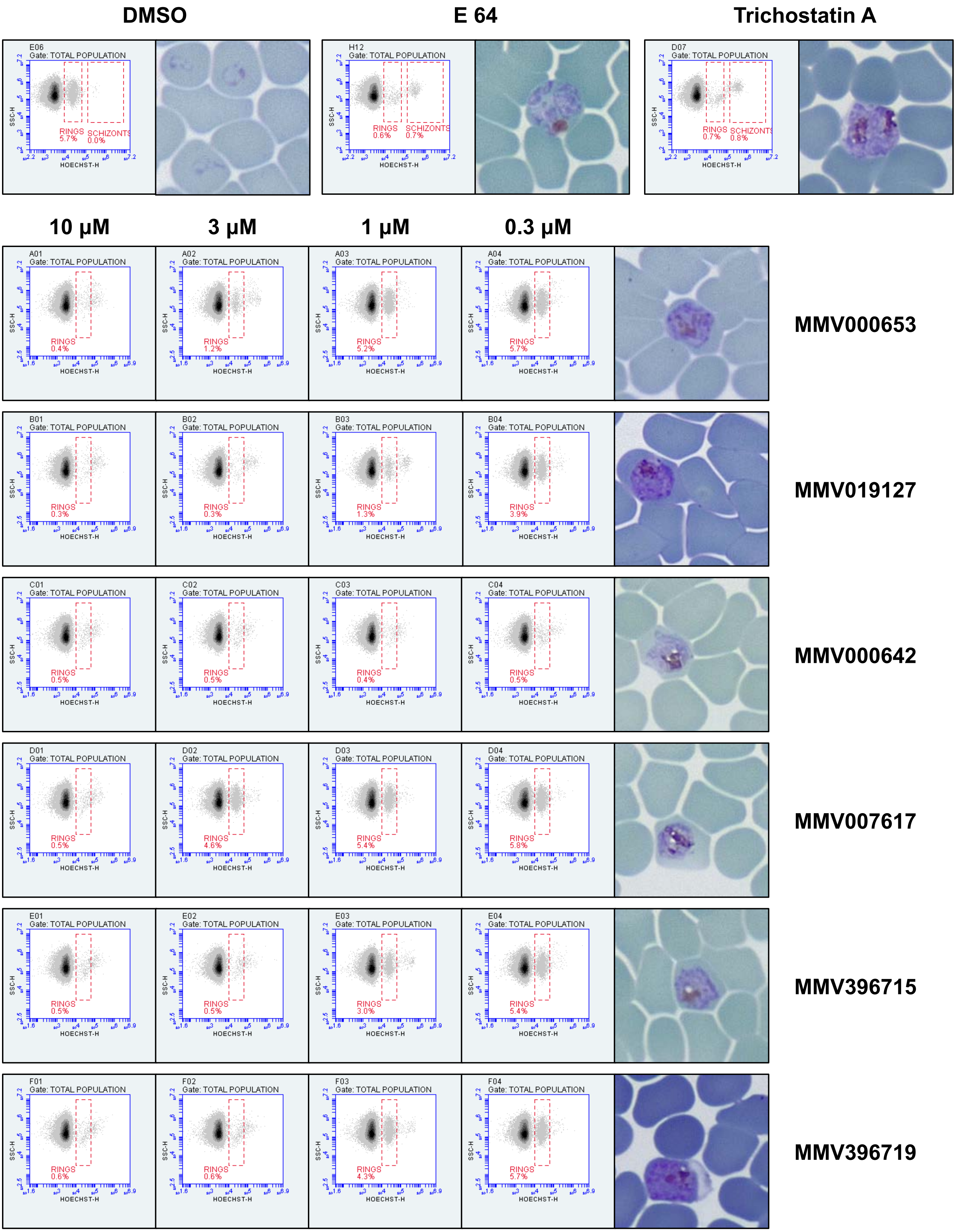

Supplementary Figure-S7 continued

10 μM | 3 μM | 1 μM | 0.3 μM

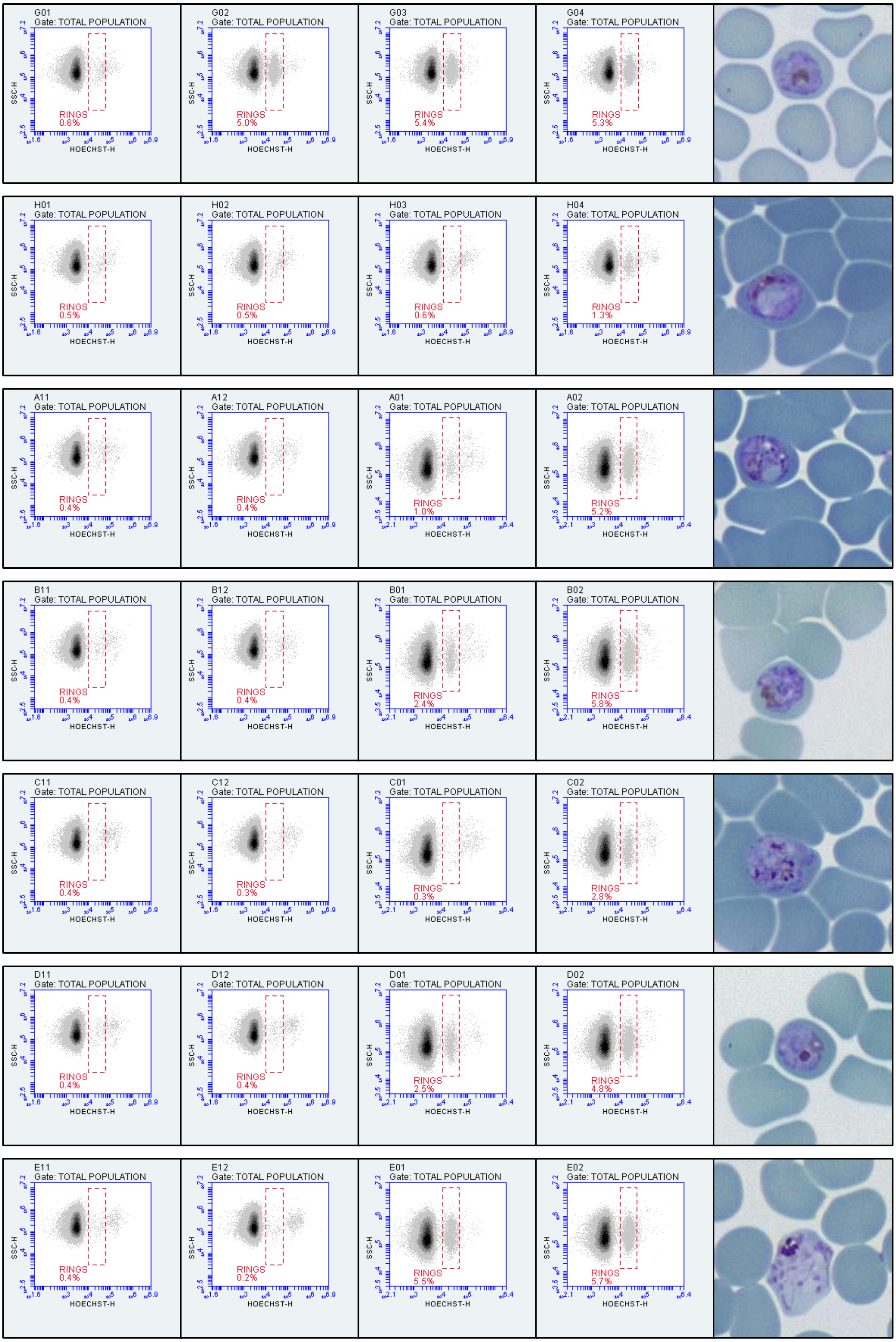

MMV666686

MMV006429

MMV396749

MMV006427

MMV007181

MMV666061

MMV019266

Supplementary Figure-S7 continued

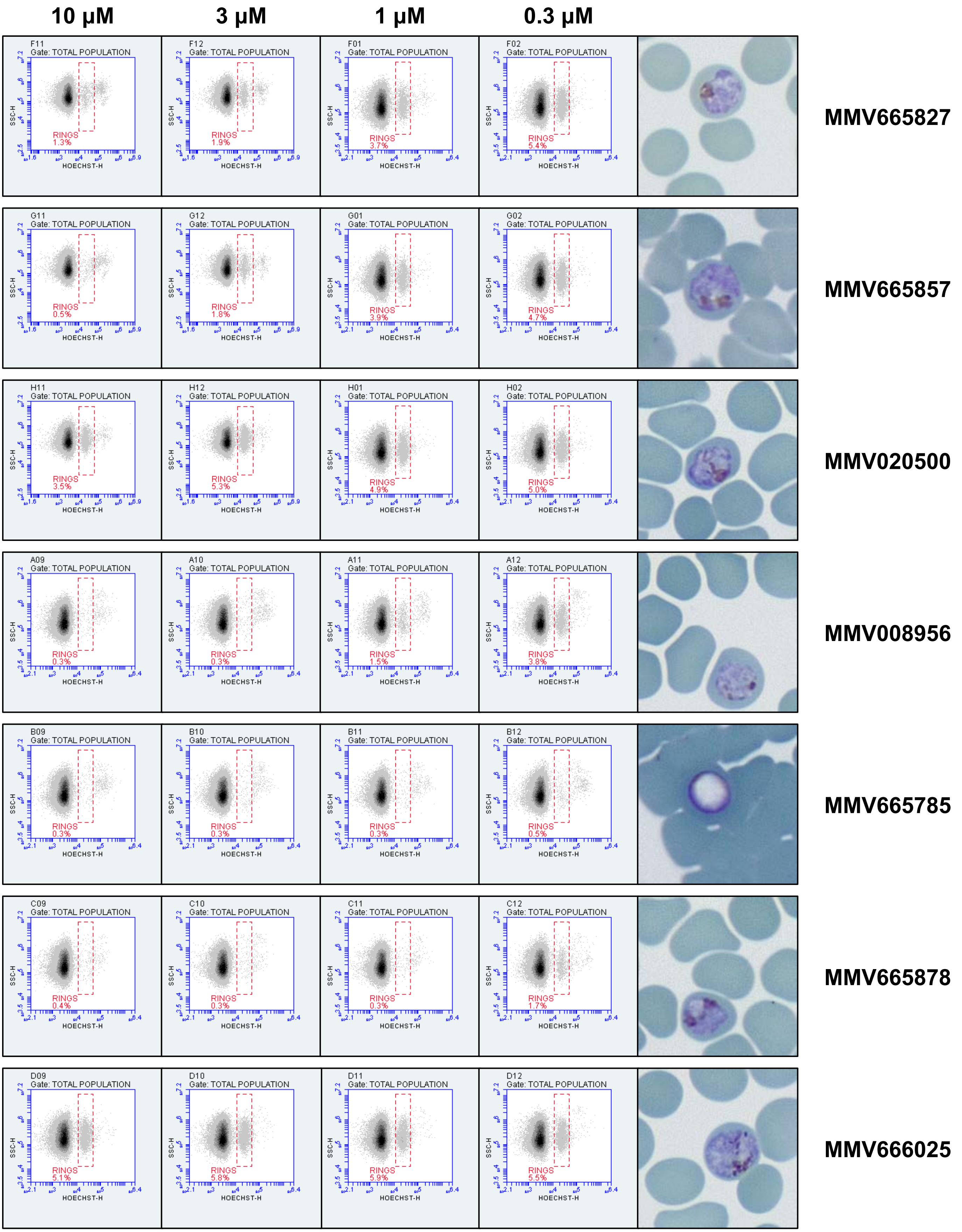

Supplementary Figure-S7 continued

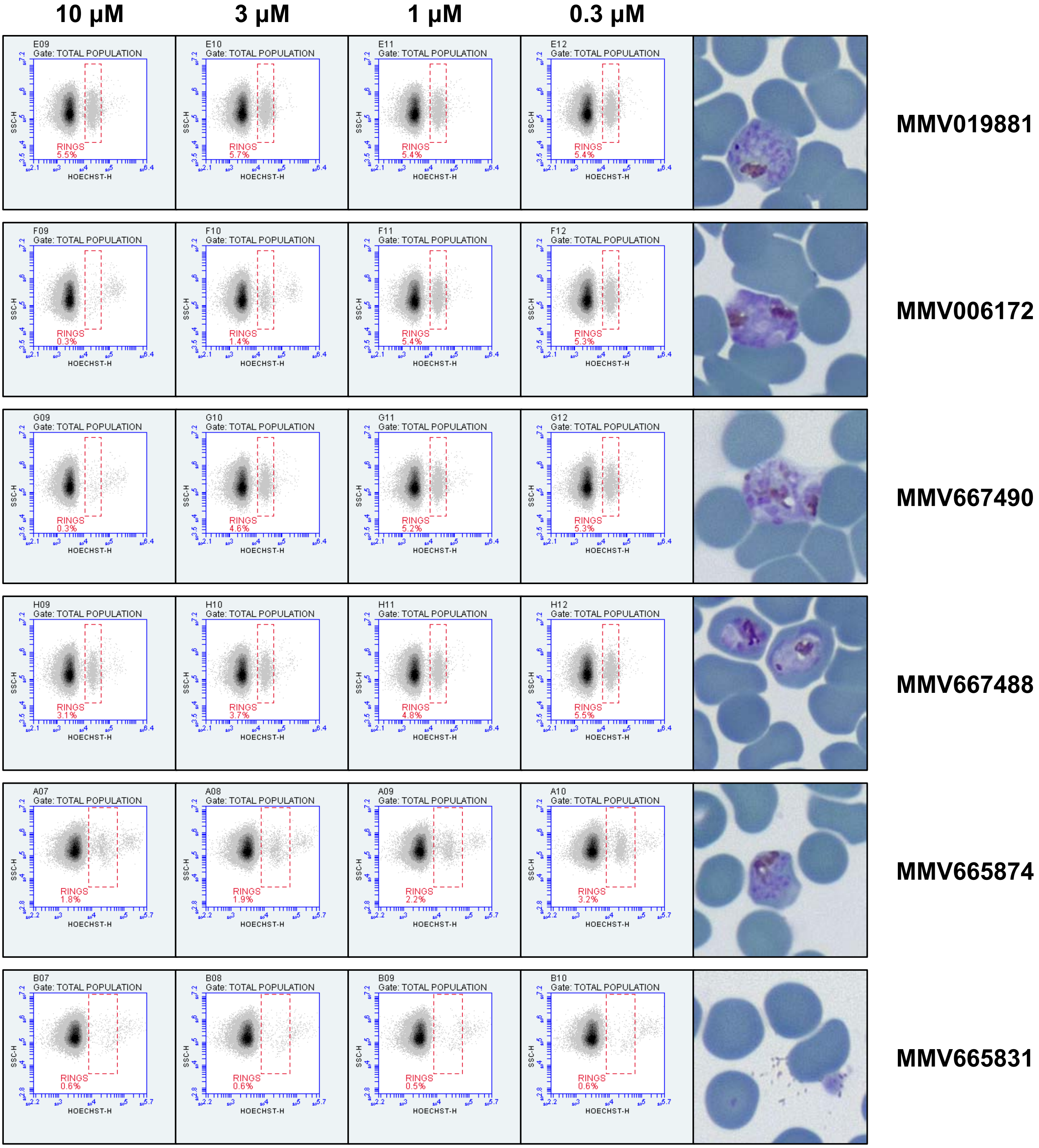

Supplementary Figure-S7 continued

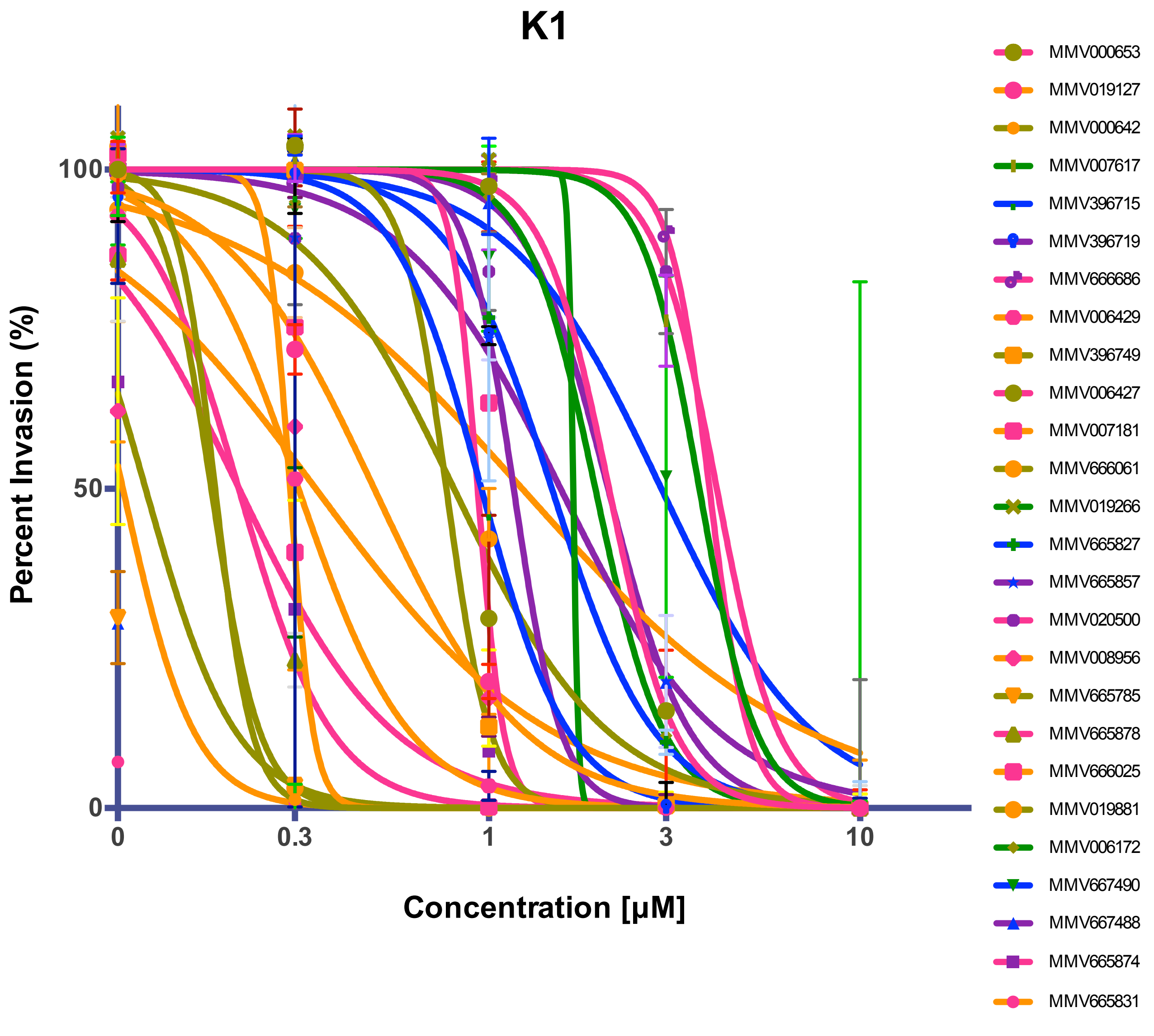

Supplement: FIG S7 [file sph001182457sf7.pdf]
