# Supplementary material for: Targeted Phenotypic Screening in Plasmodium falciparum and Toxoplasma gondii Reveals Novel Modes of Action of Medicines for Malaria Venture Malaria Box Molecules
Source: mSphere. 2018 Jan 24;3(1):e00534-17. doi: 10.1128/mSphere.00534-17 (PMC5770543; doi:10.1128/mSphere.00534-17)

Supplementary Figure-S8

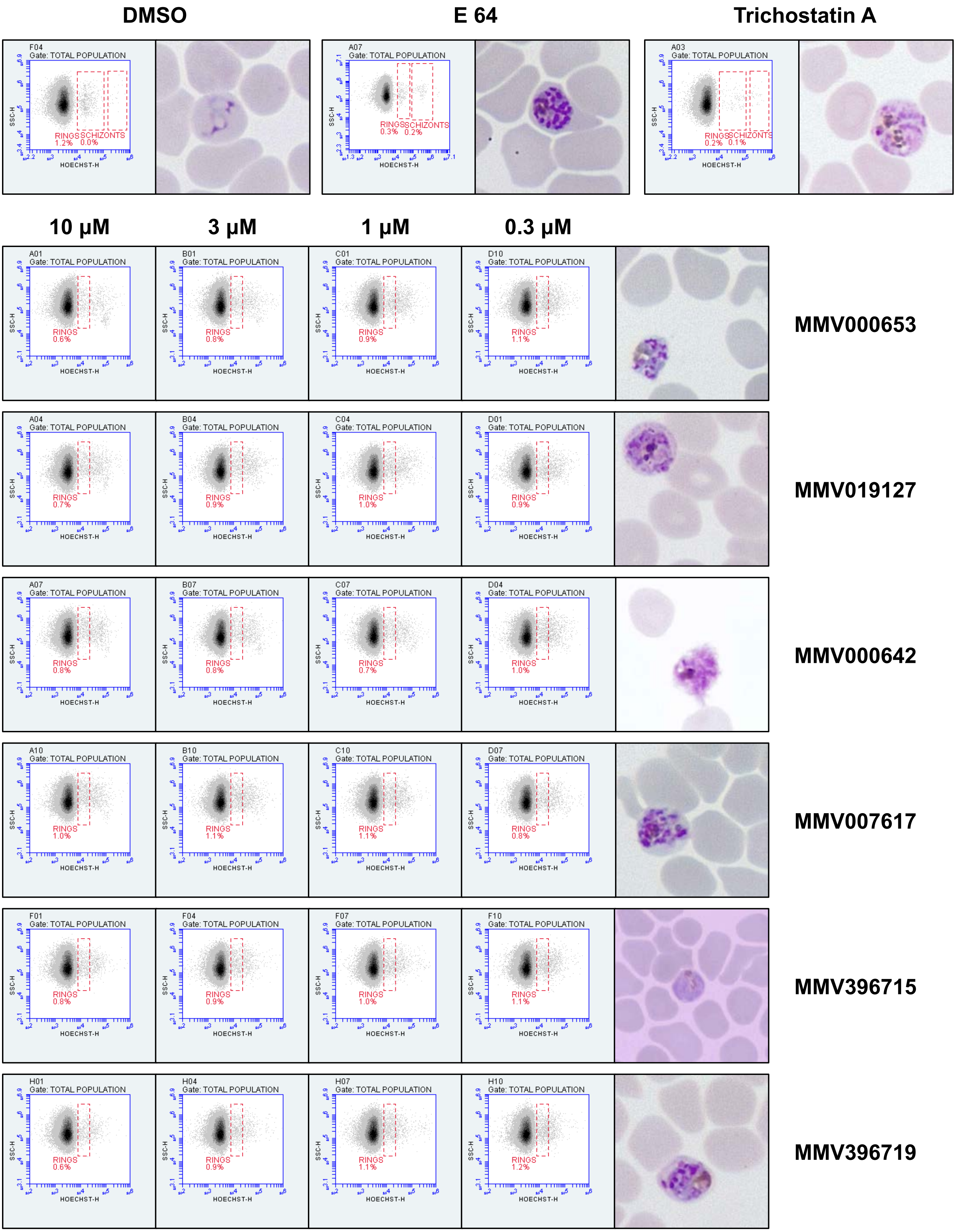

Supplementary Figure-S8 continued

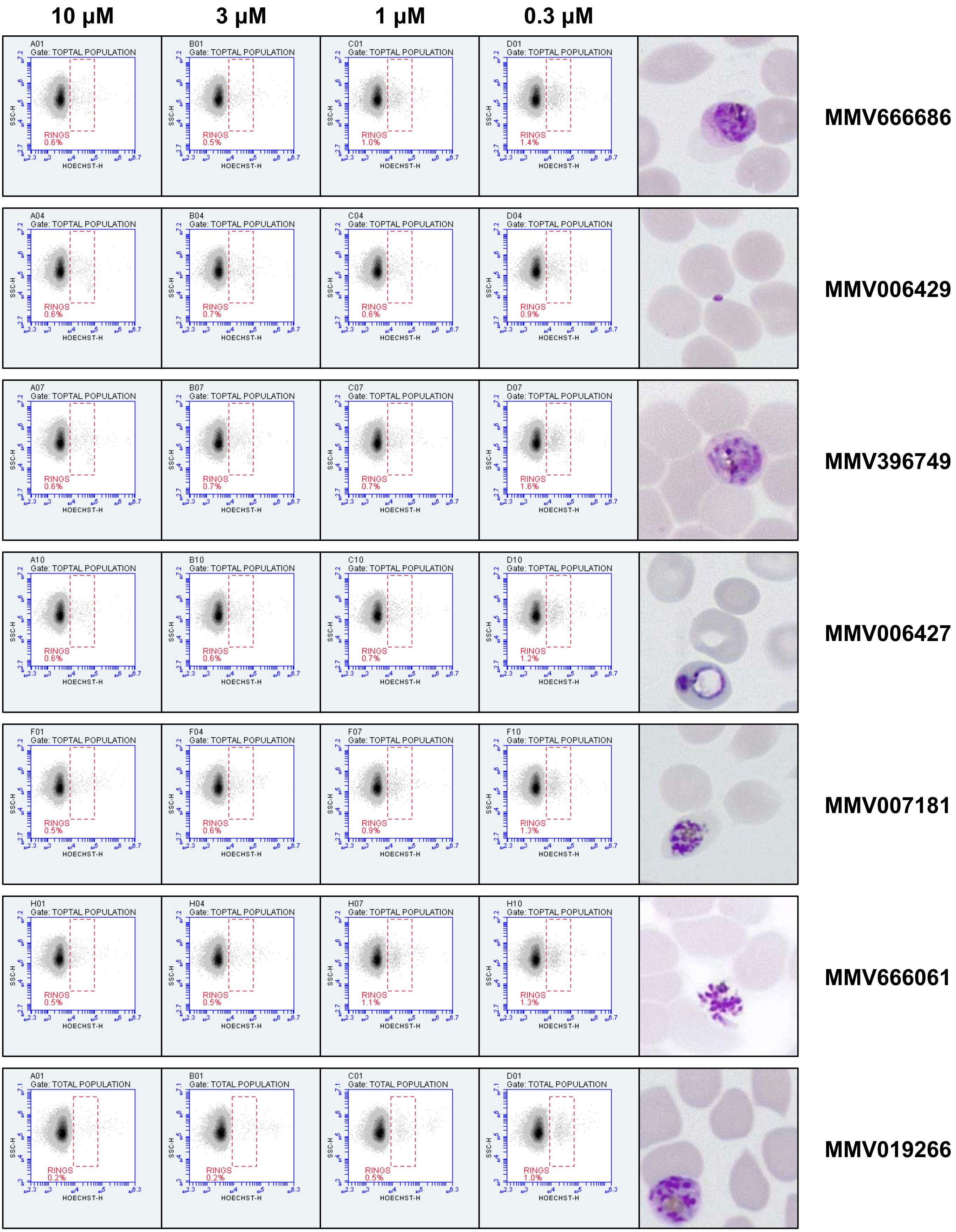

Supplementary Figure-S8 continued

10 µM | 3 µM | 1 µM | 0.3 µM

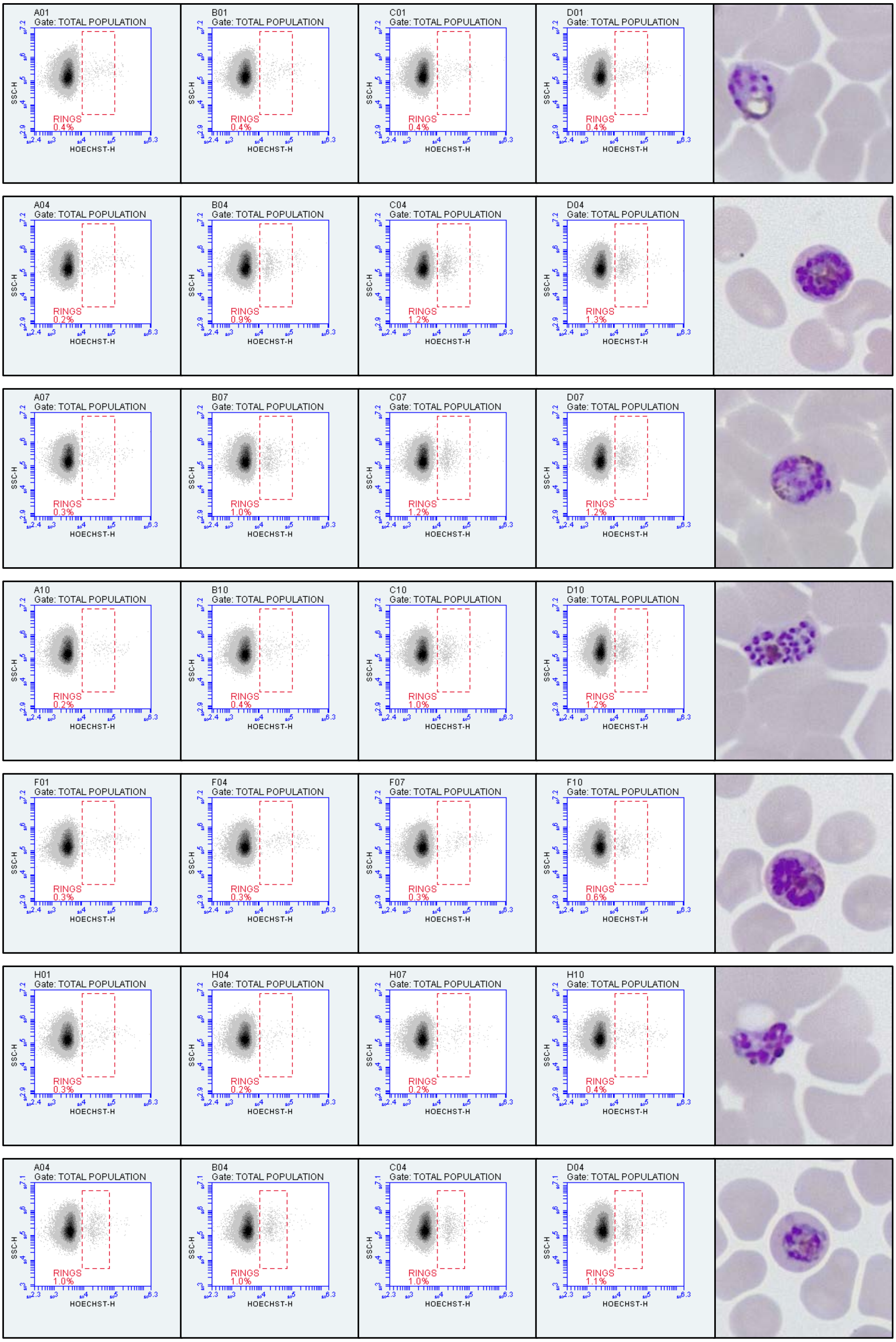

MMV665827

MMV665857

MMV020500

MMV008956

MMV665785

MMV665878

MMV666025

Supplementary Figure-S8 continued

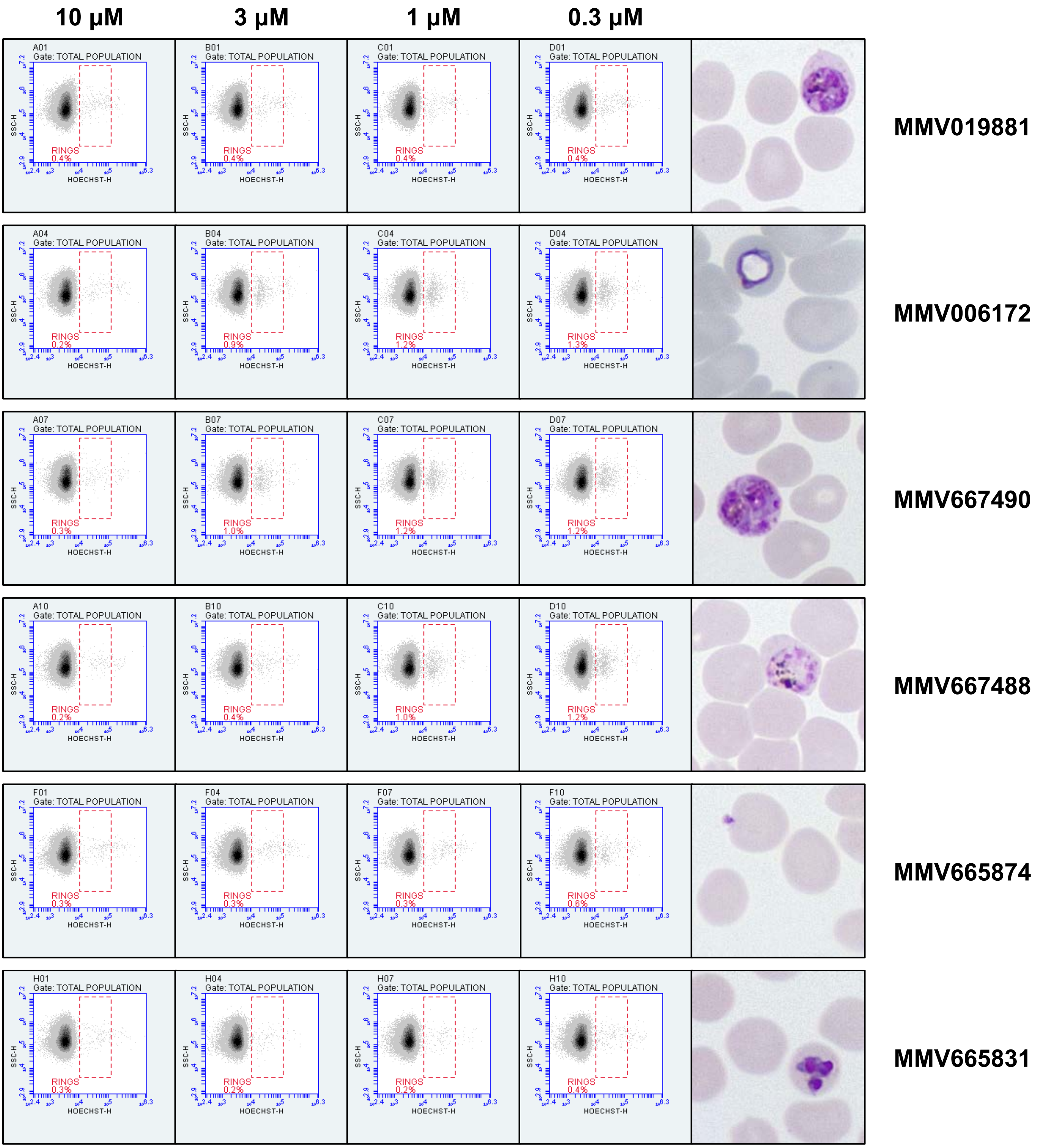

Supplementary Figure-S8 continued

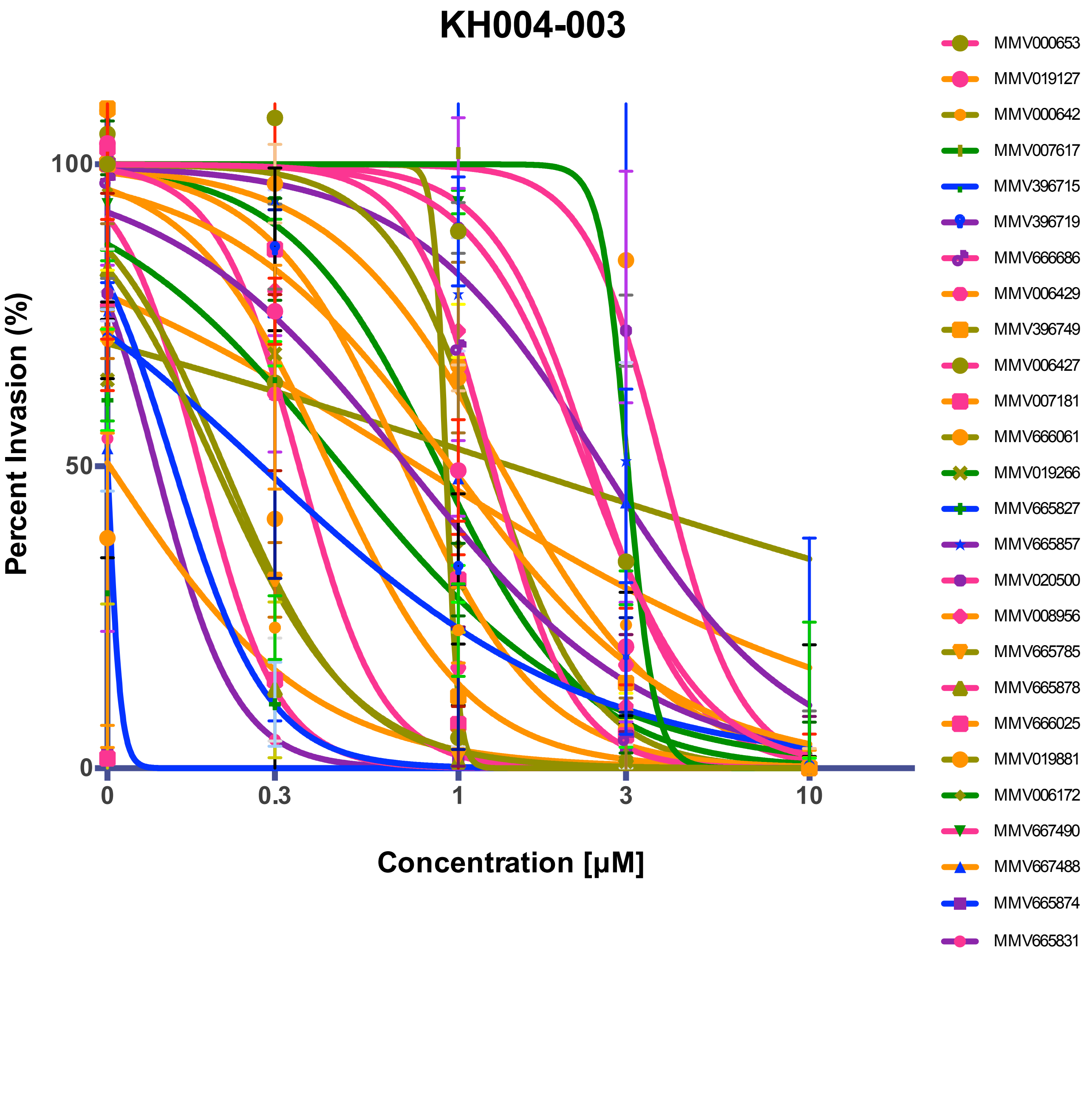

Supplement: FIG S8 [file sph001182457sf8.pdf]
